# Preferential Reduction of 2‑Phenylpyridine under D_2_: Palladium Nanoparticles Stabilized by *N*‑Heterocyclic Carbenes Prefer D_2_ to H_2_

**DOI:** 10.1021/acs.nanolett.5c04736

**Published:** 2026-04-10

**Authors:** Oscar Suárez-Riaño, Daniel Esteban Galvis-Sandoval, Gabriel Mencia, Javier Navarro-Ruiz, Stella Christodoulou, Nicolas Ratel-Ramond, Pier-Francesco Fazzini, Simon Tricard, Iker Del Rosal, Romuald Poteau, Edwin A. Baquero, Bruno Chaudret

**Affiliations:** ‡ Estado Sólido y Catálisis Ambiental (ESCA), Departamento de Química, Facultad de Ciencias, 28021 Universidad Nacional de Colombia, Carrera 30 No. 45-03, Bogotá 111321, Colombia; § 131805 Laboratoire de Physique et Chimie de Nano-Objets (LPCNO), UMR, 5215 INSA−CNRS−UPS, Institut National des Sciences Appliques 135, Avenue de Rangueil, 31077 Toulouse, France; ∥ 137668 CEMES−CNRS, Université de Toulouse, CNRS, 29 Rue Jeanne Marvig, 31055 Toulouse, France

**Keywords:** nanoparticles, palladium, *N*-heterocyclic carbene ligands, hydrogenation, deuterium, DFT calculations

## Abstract

Late-stage hydrogen isotope exchange and dearomatization reactions under D_2_ have recently been used for preparing labeled compounds using metal nanoparticle catalysts. In the case of dearomatization, lower rates are usually observed under D_2_ compared to H_2_ as a result of a kinetic isotope effect. Here, we report the synthesis of NHC-stabilized PdNPs, their characterization, and their use for the reduction of 2-phenylpyridine to 2-phenylpiperidine or 2-phenylpiperidine-*d*_6_, which reveals an unexpected preference for D_2_ over H_2_. This effect is observed using NPs stabilized by polyvinylpyrrolidone (Pd@PVP) but is more pronounced in the presence of NHC ligands (Pd@NHC). DFT calculations reveal that the phenomenon arises from a higher surface concentration of deuterides relative to hydrides in both systems, which is enhanced by the electronic influence of NHC ligands. This study provides the first report and mechanistic insight into an unusual isotopic effect in NP-catalyzed deuteration, highlighting the pivotal role of ligand–NP interactions.

## Graphical abstract

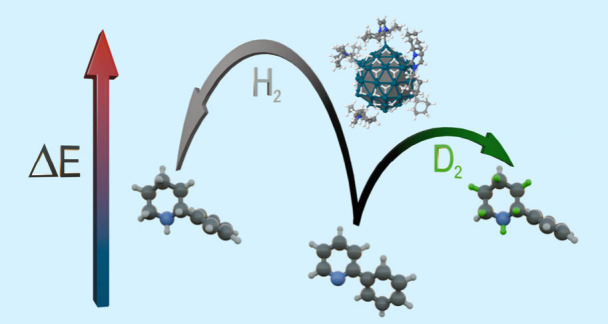

Deuterium incorporation into bioactive molecules has emerged as an effective and promising technology in chemistry and health sciences. The presence of deuterium is important for mechanistic studies, drug discovery and development, and proteomics and metabolomics.^1^ Furthermore, deuterium incorporation has recently appeared as bringing new properties to selected drugs and importantly enhanced biodisponibility. Deuterium can be incorporated by hydrogen isotopic exchange (HIE) or the addition to unsaturated substrates, leading to a reduction reaction.^2^ HIE is the most popular method since it does not change the structure of the molecule. It can be performed by using molecular catalysts, soluble nanoparticles, or heterogeneous catalysts. On the other hand, incorporating deuterium into unsaturated compounds is targeted especially for heterocycles, as in a dearomatization reaction,^3^ but using D_2_ instead of H_2_. Thus, for example, reduction of pyridines with deuterium is an alternative to obtaining deuterated piperidine derivatives directly.

We have previously shown that *N*-heterocyclic carbene (NHC)-stabilized NPs of Ru,^4^ Ir,^5^ Ni,^6^ RuIr,^7^ and PdNi^8^ were active catalysts for the HIE of a variety of substrates and exhibit different selectivities resulting from both the nature of the metal and the unique NHC ligand stereoelectronic properties: high σ donors and steric hindrance, resistance against high temperatures, great variety of substituents, among others.^9–13^ Thus, Ru NPs fully reduce 2-phenylpyridine (2PP), a convenient model substrate, under 1 atm of hydrogen or deuterium into 2-cyclohexylpiperidine,^7^ whereas Ni NPs catalyze the HIE of 2PP with a selectivity depending on the ligands and not leading to aromatic ring reduction.^6^ With regard to third row transition metals, Ir NPs lead to a surprising selectivity (deuteration at different positions from being the closer to heteroatoms),^7,14^ whereas Pt NPs were found totally inactive for HIE of 2PP in mild conditions.^15^ Similarly, Pd NPs stabilized with water-soluble NHC ligands display remarkable stability under catalytic conditions.^16,17^ Motivated by these findings and seeking new avenues to control isotopic selectivity, we turned our attention to Pd, previously shown to lead to remarkably stable nanoparticles when using water-soluble NHC ligands.^16,17^ During HIE studies with 2PP, we observed the reduction of the pyridyl ring associated with an intriguing reactivity pattern that, at first glance, resembled an unusual isotope effect, in which better conversions were obtained using D_2_ compared to H_2_. To the best of our knowledge, this effect has no precedent in hydrogenation reactions with palladium nanoparticles. Here, we present a comprehensive study of this unexpected effect. We detail the catalytic performance of NHC-stabilized Pd nanoparticles in HIE reactions, examine the influence of reaction conditions and ligand effects, and propose a mechanistic rationale for the observed D_2_/H_2_ selectivity based on detailed DFT calculations. This study not only uncovers a previously unreported facet of Pd-catalyzed deuteration but also introduces new strategies for designing nanoparticle-based isotopic labeling catalysts that may be valuable for late-stage saturation of pyridine- or phenyl-containing drug molecules to improve key pharmacological properties.^18^

Pd NPs were prepared according to modifications of a known method (see the Supporting Information), i.e., hydrogenation of an organometallic precursor, Pd_2_(dba)_3_ (dba = benzylideneacetone), under 3 bar of H_2_ at room temperature. The stabilizers were either a polymer, polyvinylpyrrolidone (PVP), a NHC-containing cyclohexyl (ICy) or mesityl (IMes) substituents at various concentrations relative to Pd, or a mesoporous carbon (C_meso_) leading to a series of NPs, namely, Pd@PVP, Pd@ICy_0.2_, Pd@ICy_0.5_, Pd@IMes_0.2_ (the number of NHC equivalents is given in the subscript compared to Pd), and Pd@C_meso_ (Figure 1a). In each case, small monodisperse and well-separated NPs were obtained. Their size was found to be 1.9 nm for Pd@PVP, 1.7–1.9 nm for NHC-stabilized NPs depending on the ligand and its concentration, and 2.6 nm for Pd@C_meso_. The particles were characterized by TEM and XRD. In addition, FTIR and solid-state NMR measurements were performed before and after reaction with ^13^CO to test the NP surface. Solid-state ^1^H–^13^C CP-MAS NMR demonstrate the coordination of the NHC ligand to the Pd surface together with the availability of the surface for further coordination (see the Supporting Information).

**Figure 1:**
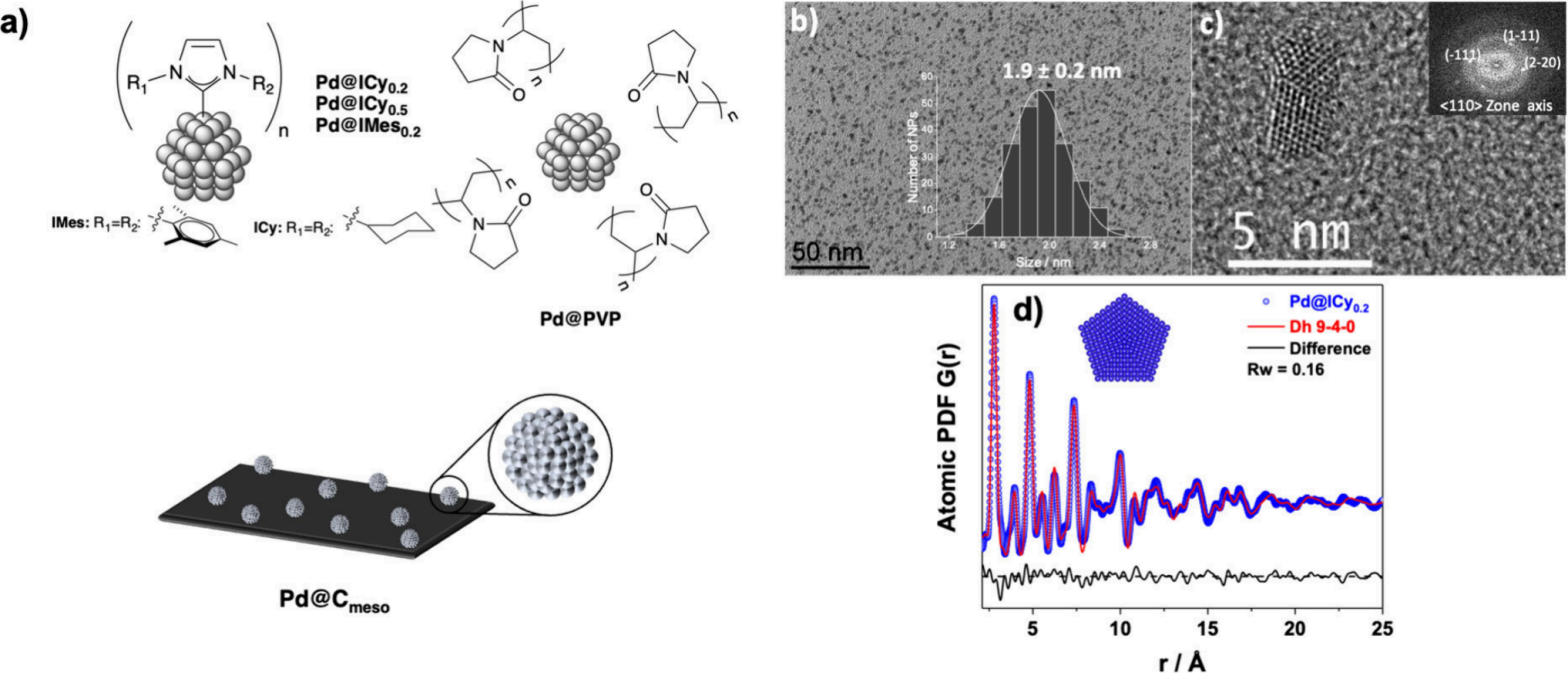
(a) Pd NPs stabilized by PVP, NHC (ICy and IMes), and mesoporous carbon synthesized in this work, (b) TEM (inset: size distribution), (c) HR-TEM micrographs (inset: FFT analysis) for Pd@ICy_0.2_ NPs, and (d) refinement of the PDF calculated from a decahedron structural model against an experimental PDF of Pd@ICy_0.2_ NPs.

TEM micrographs displayed well-dispersed and homogeneous ultrasmall PdNPs (1.7 and 1.9 nm) (Figure 1b and Figures S6, S7, and S10). XRD measurements of the Pd@ICy NPs were not conclusive due to their small size (Figure S4). However, HR-TEM images with fast Fourier transformation (FFT) analysis provide evidence of the fcc monocrystalline nature of the PdNPs (Figure 1c), and EDX analysis provides evidence of the presence of Pd (Figure S11). WAXS measurements were performed for Pd@ICy_0.2_ and Pd@IMes_0.2_ NPs (Figure 1d and Figure S5). They confirmed the fcc crystalline structure of the PdNPs, with correlation lengths between 1.8 and 2.0 nm, which is in good agreement with the sizes observed by TEM and HR-TEM.

Pd@PVP and Pd@ICy were tested as nanocatalysts in the reaction of D_2_ with 2PP as a model substrate at 55 °C under 2 bar of D_2_ for 24 h (Table 1). First, we observed a selective reduction of the pyridyl group, unexpected in these conditions,^19^ giving rise to 2-phenylpiperidine-*d*_6_ (see the Supporting Information for spectral elucidation). Several conditions were used for this reduction reaction under D_2_. As shown in Table 1, the best reaction conditions were 90 °C, 24 h, and 4 bar of D_2_ for Pd@PVP (Table 1, entry 3). Pd@ICy_0.2_ was found to be a more active catalyst than Pd@PVP (Table 1, entries 9 and 1, respectively). It may result from a positive electronic effect of the NHC ligands in catalysis compared to PVP, likely due to their highly σ-donating character. Ligand effects have been previously observed in other MNPs.^7,20^ Moreover, the presence of a higher concentration of NHCs on the surface is harmful for the catalytic performance, presumably for steric reasons (Table 1, compare entries 9, 11, and 12). A higher ligand coverage inhibits the substrate coordination to the surface, so that no reaction was observed when Pd@ICy_1.0_ was used as a nanocatalyst (Table 1, entry 12). The molecular precursor Pd_2_(dba)_3_ did not lead to any dearomatization (Table 1, entry 15), confirming that the Pd molecular species are not able to catalyze the reduction of aromatic rings.

**Table 1:**
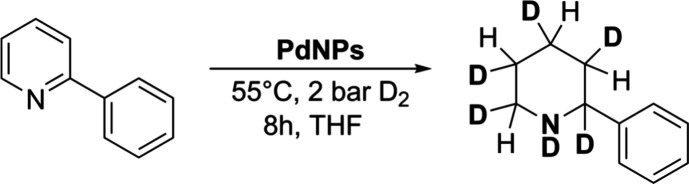
Dearomatization Reaction of 2-Phenylpyridine Using Different PdNPs as Nanocatalysts^a^

entry	catalyst	temperature (°C)	pressure D_2_ (bar)	yield (%)^b^
1	Pd@PVP	55	2	41.5
2		55	2 (H_2_)	27.0
3		90	4	>99
4		90	4 (H_2_)	>99
5	Pd@C_meso_	55	2	78.2
6		55	2 (H_2_)	50.0
7^c^		55	2	42
8^c^		55	2 (H_2_)	28
9	Pd@ICy_0.2_	55	2	50.2
10		55	2 (H_2_)	15.2
11	Pd@ICy_0.5_	55	2	36.9
12	Pd@ICy_1.0_	55	2	
13	Pd@IMes_0.2_	55	2	83.1
14		55	2 (H_2_)	41.9
15	Pd_2_(dba)_3_	55	2	

a Pd (Pd_2_(dba)_3_) (5 mol % ), Pd (Pd@PVP) (8 mol %), Pd (Pd@NHC) (5 mol %), Pd (Pd@C_meso_) (7 mol %), 2-phenylpyridine (0.1399 mmol) during 24 h, and THF (5 mL).

b Determined by NMR.

c The reaction for 8 h using 1,3,5-trimethoxibenzene as the internal standard.

Interestingly, when we performed the reaction using H_2_ as a reaction gas while operating under the same conditions, we observed a very intriguing effect. Thus, we found that the reaction proceeded better with D_2_ than with H_2_ for Pd@ICy_0.2_ (Table 1, entries 9 and 10, and Figure S19), Pd@PVP (Table 1, entries 1 and 2, and Figure S20), and Pd@IMes_0.2_ (Table 1, entries 13 and 14, and Figure S23). Furthermore, note that this unusual isotopic effect was more pronounced using NHC-stabilized NPs rather than Pd@PVP.

To determine the origin of this apparent inverse isotopic effect, we first examined the generality of this observation. PdNPs stabilized with another NHC ligand (IMes) Pd@IMes_0.2_ led to a reduction reaction displaying 83.1 and 41.9% yields using D_2_ and H_2_, respectively (Table 1, entries 13 and 14, and Figure S23). In addition, PdNPs supported on mesoporous carbon (Pd@C_meso_) were used and found to be even more active in the reduction of 2-PP compared to Pd@PVP and Pd@ICy_0.2_ (Table 1, compare entries 5, 9, and 1, Table S2, and Figure S21). This enhanced activity can be attributed to improved substrate accessibility to the Pd surface in Pd@C_meso_, in contrast to the sterically hindered surface characteristic of the Pd@PVP system. Under optimized conditions for Pd@C_meso_ (70 °C, 12 h, and 4 bar D_2_/H_2_), no significant difference in the yield was observed between D_2_ and H_2_ (Table S2, entries 1–8), although prolonged reaction times or higher temperatures led to competitive reduction of the phenyl ring (Figure S22). In contrast, at a lower temperature (55 °C) and shorter reaction time (8 h), Pd@C_meso_ again displayed an inverse isotopic effect, with ^1^H NMR integration revealing higher product yields when D_2_ was used as the reducing gas (Table 1, entries 7 and 8, and Figure 2a and b). The corresponding ^2^H NMR spectra (Figure 2c and Figure S24) provide direct evidence of deuterium incorporation and confirm selective reduction of the pyridine ring without H/D exchange. Furthermore, in order to understand the difference in catalytic activity between Pd@C_meso_ and Pd@ICy_0.2_, we used 2,2′-bypyridine and biphenyl as substrates, obtaining full conversion to 2,2′-bypiperidine-*d*_12_ and cyclohexyl-*d*_6_ benzene with Pd@C_meso_ (Figures S25 and S26), whereas 2,2′-bipyridine was slightly reduced and biphenyl did not react when using Pd@ICy_0.2_ (Figures S27 and S28). These observations demonstrated that, for Pd@ICy_0.2_, a directing group is needed, in this case, nitrogen in the pyridine ring, while Pd@C_meso_ is more active, as its surface is more available due to the absence of NHC ligands.

**Figure 2:**
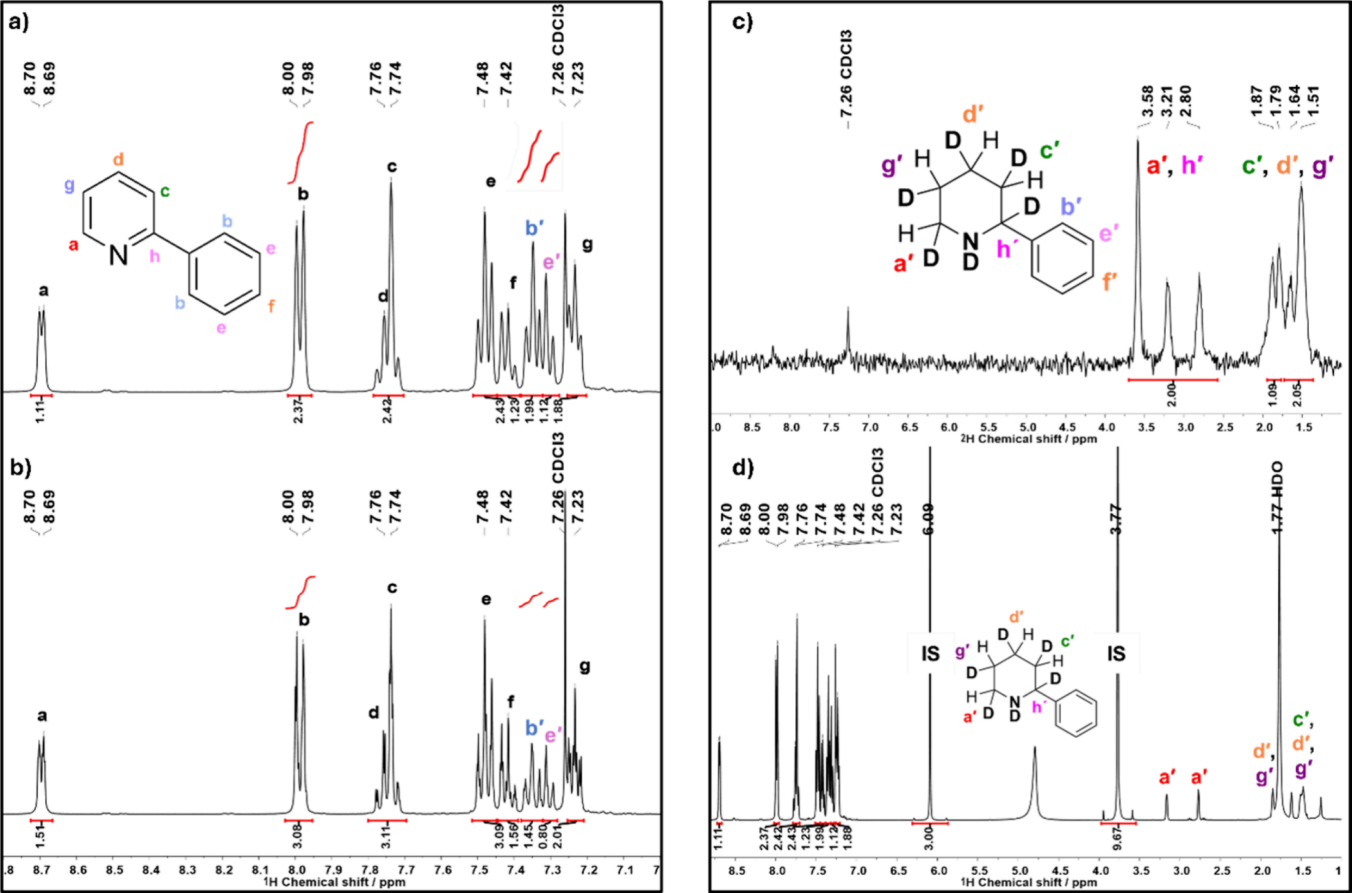
^1^H NMR spectrum (400 MHz, CDCl_3_) of the 2-phenylpyridine dearomatization reaction using Pd@C_meso_ (8 h, 55 °C, and 7 mol % Pd) under (a) 2 bar D_2_ and (b) 2 bar H_2_, (c) ^2^H NMR (61 MHz, CHCl_3_) spectrum of the reaction performed under 2 bar D_2_, and (d) full ^1^H NMR spectrum (400 MHz, CDCl_3_) of the 2-phenylpyridine dearomatization reaction performed under 2 bar D_2_. IS denotes the signals attributed to the internal standard used (1,3,5-trimethoxybenzene).

It therefore appears that the reaction is specific to Pd, as with other different M@NHC NPs, reactions with D_2_ resulted in the expected H/D exchange^20^ or to the full reduction of both aromatic rings (Ru@NHCs) and that the presence of ligands, specifically NHC ligands, on the PdNP surface increases the difference of the reduction rate between D_2_ and H_2_. In order to understand the origin of this surprising effect, DFT calculations were carried out.

The previous results prompted us to perform DFT calculations to explain the unusual isotopic effect. Pd@PVP and Pd@ICy_0.2_ NPs were modeled as fully hydrogenated small nanoclusters made of 55 Pd atoms, with a diameter of the metal part of about 1.1 nm, in good compromise between accuracy and computational cost, although it is slightly smaller than the experimental counterpart. In the present work, the metallic core is modeled as a 55 atom icosahedron, consistent with morphologies that have been experimentally observed or computationally proven to be stable (also see the discussion in the Supporting Information).^21–23^ Pd@PVP is represented by a hydrogenated Pd_55_ cluster, as PVP is not explicitly considered due to its weak or non-coordinating character. To model Pd@ICy_0.2_, five ICy ligands were grafted onto the surface of the hydrogenated Pd_55_ cluster, in agreement with the experimental ICy surface coverage (see the Supporting Information). Two representative structures are shown in Figure S30. Additionally, models with lower ICy surface coverage, namely, Pd_55_(X)_*n*_ICy_3_ (X = H or D), were also considered. These models proved particularly useful for efficiently exploring thermodynamic, vibrational, and kinetic properties as a function of the H (respectively D) surface composition within DFT calculations.

The first hydrogen/deuteride insertion into 2PP was examined, to assess whether it could be the subject of an inverse KIE. Given previous studies^24–26^ on hydrogen isotopic exchange on various *N*-heterocycles catalyzed by RuNPs,^14^ this reaction is assumed to proceed via N coordination of 2PP at the catalyst surface, thereby facilitating the first hydrogenation step at the carbon atom in the α position relative to the nitrogen atom. This reaction, which will be later described at the atomic scale, can be summarized by

$$2\mathrm{PP}\left(g\right)+\mathrm{PdNP}\to 2{\mathrm{PP}}^{*}\to 2\mathrm{PP‐}X^{*}$$

where * denotes adsorption on the nanoparticle surface and X represents either H or D. The adsorption of 2PP onto the Pd surface is exothermic by approximately 20 kcal mol^–1^, regardless of the specific model considered (*vide infra*). We assume this step to be barrierless provided that sufficient space is available on the nanoparticle surface. The first hydrogenation proceeds via a transition state, hereafter referred to as TS1. Considering the arguments in ref 27 (see also the short introduction in the Supporting Information), it is informative to calculate the Δ_CH/CD_^⧧^/Δ_MH/MD_ ratio for both systems, with the same relevant and representative 1.3 H/Pd_surf_ hydrogen coverage value, corresponding to 56 hydrogens, slightly above the *p* = 1 bar and rt optimal coverage value found in ref 22. For Pd_55_(μ_3_-H)_56_ and the simplified Pd_55_(μ_3_-H)_56_(ICy)_3_ model, we find that the Δ_CH/CD_^⧧^/Δ_MH/MD_ ratio is significantly lower than 1, with a value of approximately 0.7 for both systems. This result suggests that no inverse KIE is expected for this reaction under reasonably realistic pressure and temperature conditions. Moreover, no collective effect arising from the three NHC ligands appears to significantly influence this ratio.

We then investigated the surface composition of the Pd_55_X_*n*_ and Pd_55_X_*n*_ICy5 models (X = H or D) under realistic temperature and pressure conditions, by using the *ab initio* thermodynamic method.^28–30^ H and D surface composition diagrams are reported in Figure 3. Red crosses indicate two experimental conditions used for dearomatization reactions, namely, *T* = 55 °C and *p*_X_2__ = 2 bar and *T* = 90 °C and *p*_X_2__ = 4 bar (see Table 1). A complete list of all surface compositions considered for this phase diagram determination is provided in the Supporting Information. Figure 3a, the diagram showing the domains of stability of hydrides on Pd@PVP NPs, can be directly compared to the stability diagram reported in ref 23 The two diagrams are overall similar: we agree that, under standard conditions, the optimal surface coverage is 1.3 H/Pd_surf_ (Pd_55_H_56_, orange area), but two main differences emerge. First, the 1.24 H/Pd_surf_ composition (Pd_55_H_52_, cyan area), which was not considered in the previous study, appears as a stable configuration in our case. Second, under high H_2_ or D_2_ pressures and low-temperature conditions, our study shows that Pd nanoparticles can begin to host subsurface hydrides or deuterides in octahedral sites (Pd_55_H_60_, pale yellow area), a feature not observed in earlier work.

**Figure 3:**
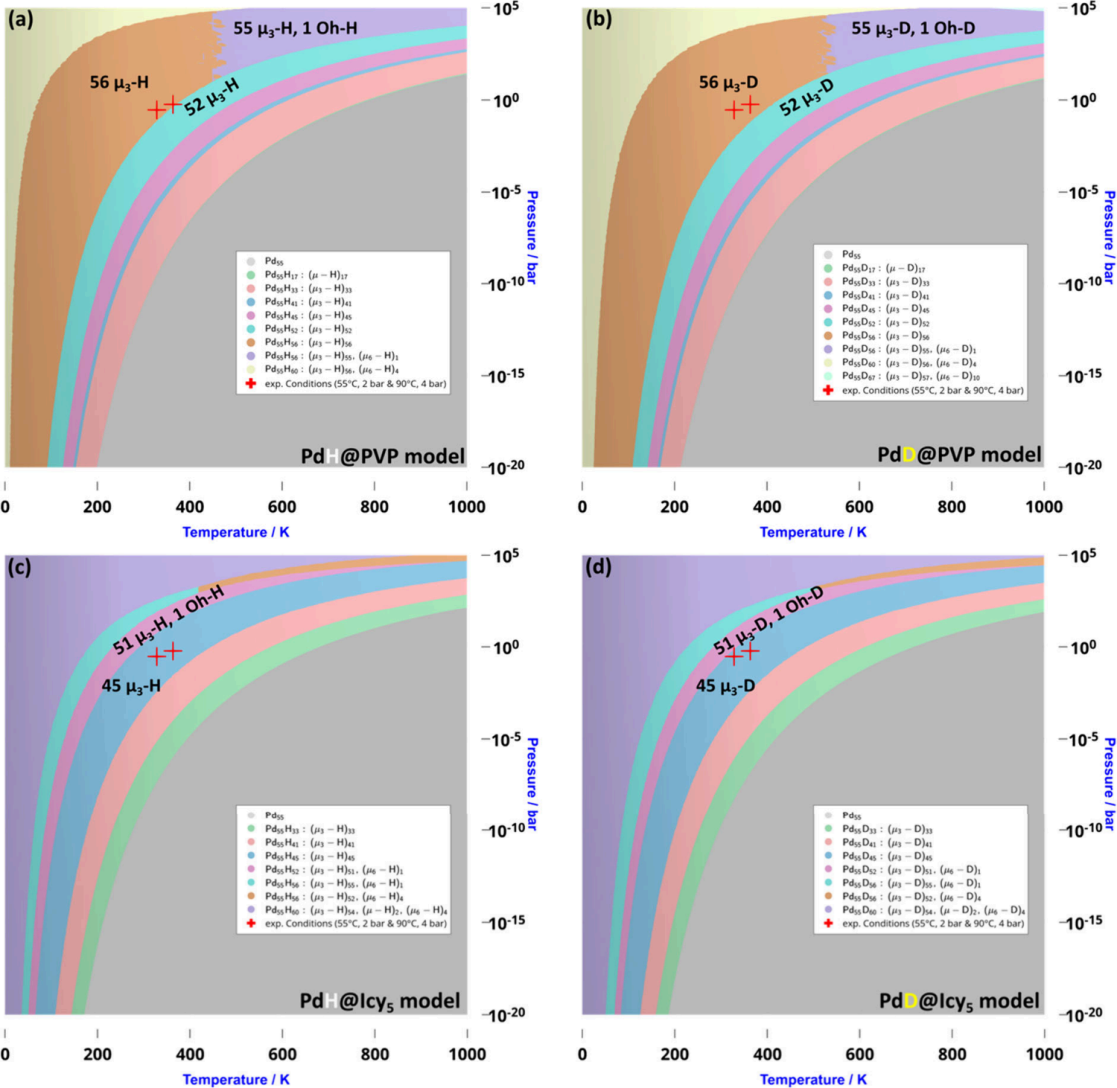
Δ*G*_ads_(*T*, *p*_H_2__) stability diagram for adsorption of (left )­H_2_(gas) and (right) D_2_(gas) on the (a and b) Pd@PVP model and (c and d) Pd@ICy_5_ model (see the Supporting Information for details). *T* ranges from 0 to 1000 K, and *p*_X_2__ ranges from 10^–20^ to 10^5^ bar. Red crosses indicate selected experimental conditions used in dearomatization reactions (see Table 1).

At first glance, the four stability diagrams in Figure 3 appear to be similar. Let us first examine the PVP case. Under the experimental conditions used in dearomatization reactions, two surface compositions are in thermodynamic competition: 1.33 H/Pd_surf_ (Pd_55_H_56_) and 1.24 H/Pd Pd_surf_ (Pd_55_H_52_). While the red crosses in Figure 3a lie very close to the lower coverage domain (1.24 H/Pd Pd_surf_), Figure 3b clearly indicates that, for D_2_, a higher surface coverage (1.33 D/Pd Pd_surf_) becomes significantly more favorable under similar conditions. As for the ICy case, similar trends are observed: under dearomatization conditions, Pd_55_ICy_5_ can host 45 surface hydrides (Figure 3c), whereas in the case of deuterides, there is a competition between this low surface coverage and a higher one, involving 51 surface deuterides and one deuterium atom in an octahedral site (Figure 3d), with this subsurface D possibly contributing to the observed difference.

Although these are relatively subtle trends, they may nonetheless provide a thermodynamic basis for the isotope effect observed in the dearomatization reactions: under identical experimental conditions, the surface and subsurface coverage and, thus, the local environment of the active sites differ between H and D, potentially impacting the reaction rate and selectivity in a small but measurable way, as observed experimentally in Table 1. We now turn to a mechanistic investigation to evaluate how higher versus lower H (or D) surface coverages can influence the activation barrier and the thermodynamics of the first insertion step.

The mechanistic study is summarized in Figure 4. Two surface compositions were considered, 1.07H (or D) per Pd_surf_ (IC-045H model, burgundy profile) and 1.33H (or D) per Pd_surf_ (IC-056H model, red profile), to evaluate whether a lower surface coverage could affect the reaction. 2PP binds strongly to the Pd surface and then reacts with a hydride/deuteride. In the transition state (TS1), the hydride shifts from μ_3_ to a terminal coordination, approaching the carbon atom of 2PP. This yields hydrogenated intermediate C_1_H, which is less stable due to a loss of conjugation, making the process slightly endothermic. Surface coverage mainly affects the barrier height (≈10 vs 20 kcal mol^–1^), while H/D substitution itself shows little direct kinetic isotope effect (see a more detailed discussion in the Supporting Information). It is rather a surface coverage difference that could account for the apparent KIE on the dearomatization of 2-phenylpyridine observed with Pd NPs.

**Figure 4:**
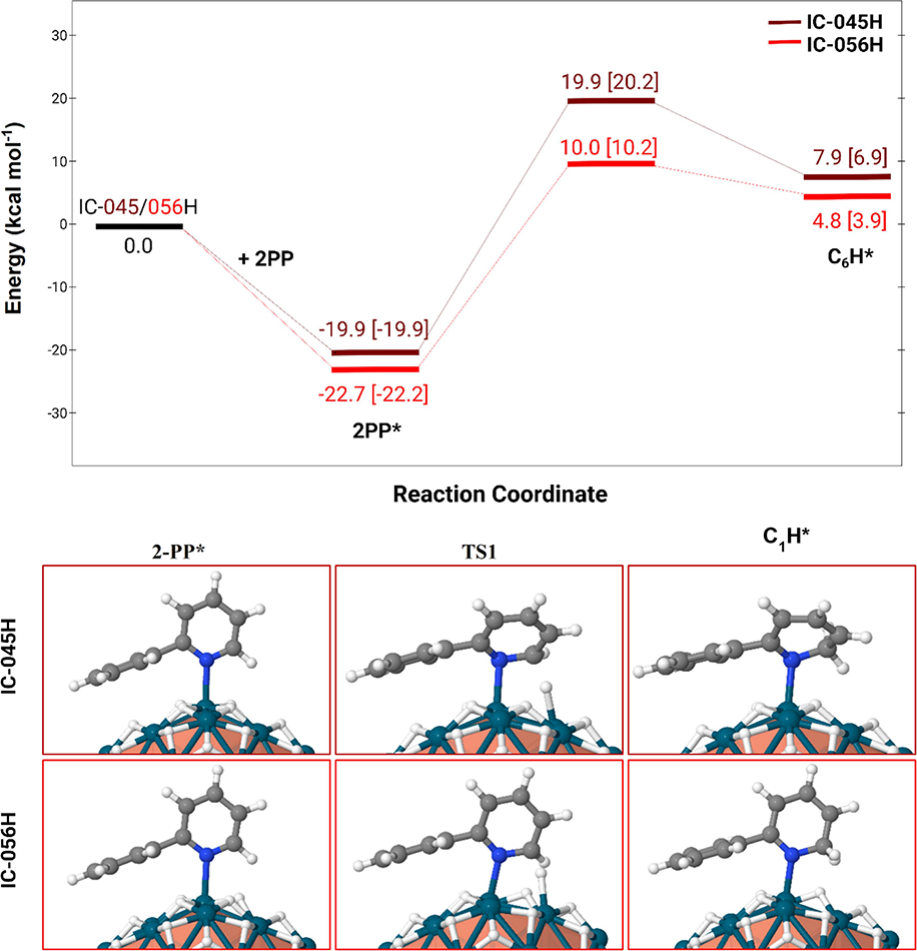
Reaction energy profile of the first hydride or deuteride insertion at carbon in the α position to nitrogen of 2-phenylpyridine. Burgundy/red: on Pd_55_H_45_ and Pd_55_H_56_, respectively. Energies are given in kcal/mol. D insertion energies are given in square brackets. The * stands for “adsorbed”.

This mechanistic study does not account for any potential direct electronic influence of the ICy ligands on the first insertion step, which could contribute to the stronger inverse KIE observed in the ICy case. The presence of ICy in the vicinity of 2PP* in a Pd_55_H_56_ICy_3_ model reduces the activation barrier for the first insertion step by about half (∼4 kcal mol^–1^ vs ∼10 kcal mol^–1^ without Icy), with no significant isotope effect at the transition state TS1, shown in Figure 5. The resulting C_1_X intermediate (H or D) is also slightly stabilized in the presence of ICy, highlighting that such ligands directly facilitate hydride/deuteride insertion beyond surface coverage effects (energies are given in the Supporting Information).

**Figure 5:**
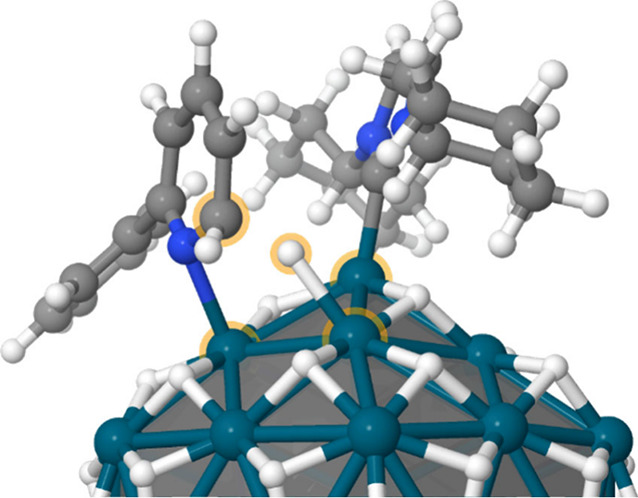
TS_1_ in the Pd_55_H_56_ICy_3_ model, where a 2PP* substrate was positioned near an ICy ligand. Atoms of the reaction site are highlighted with an orange halo.

Under the same experimental conditions, such as *p*_D_2__ = 2 bar and *T* = 55 °C, Pd@ICy_0.2_ nanoparticles exhibit slightly higher activity in the dearomatization of 2-phenylpyridine compared to Pd@PVP. This observation contrasts somewhat with the thermodynamic stability diagrams presented in Figure 3, which indicate a slightly higher surface deuteride coverage for Pd@PVP, suggesting that PVP-stabilized nanoparticles should, in principle, be more efficient. This apparent contradiction may be resolved by considering a synergistic effect in Pd@ICy_0.2_: the possible presence of subsurface deuterides, absent in Pd@PVP, and the direct electronic influence of ICy ligands may together enhance the reactivity of the system beyond what surface coverage alone would predict.

In conclusion, it is remarkable that, for a simple reaction, such as the interaction of phenylpyridine with deuterium, very different reactivities are found for different metals, namely, no reaction with Pt, full reduction with Ru, HIE with Ni and Ir, and selective partial reduction with Pd. This emphasizes the importance of catalysts in such reactions. With palladium, we show that we can selectively introduce deuterium through the reduction of the pyridyl ring to give *d*_6_-labeled substituted piperidines. However, the most interesting aspect of this work is the apparent inverse kinetic isotope effect observed for the reduction of the pyridyl ring: the reaction proceeds more efficiently under D_2_ than under H_2_. Our mechanistic analysis attributes this effect to the higher surface coverage of deuterides relative to hydrides on NHC-stabilized Pd nanoparticles combined with the positive electronic influence of the NHC ligands, which enhances reactivity toward deuterium. Beyond its synthetic utility, this unexpected behavior highlights the transformative impact of NHC–nanoparticle interactions on the isotopic reactivity. It also demonstrates the predictive power of modeling, which now enables not only the quantification of hydride species on nanoparticle surfaces but also the anticipation of their distinct reactivities. Altogether, these findings open new perspectives for the rational design of nanoparticle catalysts in isotopic labeling and provide a foundation for the deeper exploration of isotope effects at the nanoscale.

## Supplementary Material



## Notes

General procedures, experimental details, and full characterization of Pd NPs: XRD and WAXS measurements, TEM images, EDX spectra, ATR–FTIR spectra, solid-state ^1^H–^13^C CP-MAS NMR spectra, ^1^H and ^13^C NMR spectra for deuteration reactions, mass spectra for selected deuteration reactions, and DFT calculations (PDF)

## †

This work is dedicated to the memory of Oscar Suárez-Riaño, an outstanding scientist and a dear friend, whose passion, smile, and unique ability to bring people together will always remain with us.
