# Supplementary material for: Preferential Reduction of 2‑Phenylpyridine under D2: Palladium Nanoparticles Stabilized by N‑Heterocyclic Carbenes Prefer D2 to H2
Source: Nano Lett. 2026 Apr 10;26(15):4936–43. doi: 10.1021/acs.nanolett.5c04736 (PMC13107521; doi:10.1021/acs.nanolett.5c04736)
Supplement: Supplementary file 1 [file nl5c04736_si_001.pdf]

Supporting Information

Preferential reduction of 2-phenylpyridine under D_2: Palladium Nanoparticles stabilized by N-Heterocyclic Carbenes prefer D_2 to H_2

Oscar Suárez-Riaño,[1,2,§] Daniel Esteban Galvis-Sandoval,[2] Gabriel Mencía,[2] Javier Navarro-Ruiz,[2] Stella Christodoulou,[2] Nicolas Ratel-Ramond,[1] Simon Tricard,[2] Iker Del Rosal,[2] Romuald Poteau,[2] Edwin A. Baquero,[1*] Bruno Chaudret[2*].*

[1]Estado Sólido y Catálisis Ambiental (ESCA), Departamento de Química, Facultad de Ciencias, Universidad Nacional de Colombia, Carrera 30 No. 45-03, 111321, Bogotá, Colombia.

[2]LPCNO, Laboratoire de Psique et Chimie de Nano-Objets, UMR, 5215 INSA-CNRS-UPS, Institut National des Sciences Appliques 135, Avenue de Rangueil, 31077, Toulouse, France.

[3]CEMES-CNRS, Université de Toulouse, CNRS, 29 rue Jeanne Marvig, 31055 Toulouse, France.

[§] This work is dedicated to the memory of Oscar Suárez-Riaño, an outstanding scientist and a dear friend, whose passion, smile and unique ability to bring people together will always remain with us.

Corresponding Authors: tricard@insa-toulouse.fr (S.T.), eabaquerov@unal.edu.co (E.A.B.), chaudret@insa-toulouse.fr (B.C.)

TABLE OF CONTENTS

1. General procedures and characterization techniques 3

2. Synthesis and characterization data for imidazolium salts and free carbenes 4

3. Synthesis and characterization data for Pd nanoparticles 7

4. XRD measurements for PdNPs 9

5. WAXS for PdNPs 10

6. TEM images for PdNPs 11

7. EDX spectra for PdNPs 14

8. ATR-FTIR spectra for PdNPs 14

9. Solid-State ^{1}H–^{13}C CP-MAS NMR spectra for PdNPs 15

10. NMR and Mass spectra for deuteration reactions 17

11. Dearomatization reaction 29

12. DFT calculations 31

13. References 36

1. General procedures and characterization techniques

Reagents

$Pd_2(dba)_3$ precursor was purchased from Nanomeps Toulouse, mesoporous carbon, 2-phenylpyridne, 3-phenylpyridine, 4-phenylpyridine, polyvinylpyrrolidone, biphenyl, pyridine, 2-methylpyridine, 2-methoxypyridine, 2-(trifluoromethyl)pyridine, and 1,3-bis(2,4,6-trimethylphenyl)-1,3-dihydro-*2H*-imidazol-2-ylidene (IMes) from Sigma-Aldrich, tetrahydrofuran from CARLO ERBA, 2,2'-bipyridine, from Alfa-Aesar, D_2 and CO gas from Air liquid (France), ^{13}CO gas from Eurisotop, H_2 from Hydrogen Gas Generator AVANTEC model 40H (water electrolysis). All the reagents were used without further purification.

Transmission Electron Microscopy (TEM)

MNPs were observed by TEM after the deposition of a drop of a solution of the isolated NPs suspended in THF over a copper grid coated with amorphous carbon. TEM analyses were performed at Raimond Castaing Microanalysis Centre (UAR 3623) (Toulouse, France) using a JEOL JEM 1400 electron microscope working at 120 kV. The NPs average size approximation was made by manual analysis of the magnified micrographs measuring 200 particles on a given grid using ImageJ software.

High Resolution TEM coupled to Energy Dispersive X-ray Spectroscopy (HR-TEM-EDX)

MNPs were observed by HR-TEM after the deposition of a drop from a solution of the isolated MNPs suspended in THF or water over a holey carbon-coated copper grid. HR-TEM analysis was performed at Raimond Castaing Microanalysis Centre (UAR 3623) (Toulouse, France). TEM and scanning transmission electron microscopy (STEM) studies were performed using a JEOL cold-FEG JEM-ARM200F operated at 200kV equipped with a probe Cs corrector reaching a spatial resolution of 0.078 nm. EDX spectra were recorded on a JEOL CENTURIO SDD detector.

Thermogravimetric analysis (TGA)

TGA analyses were performed in a TGA/DSC 1 STAR System equipped with an ultra-microbalance UMX5, a gas switch GC200 and sensors DTA and DSC. The samples were analyzed through a two steps oxidation/reduction method. First the sample was heated from 25 °C to 500 °C at 20°C/min under air and kept at 500 ºC during 2h. After cooling down, it was heated again from 25 °C to 700 °C at 30 °C/min under a gas mixture Ar/H_2 4% and kept at 700 ºC during 3h.

Attenuated Total Reflection Fourier Transform Infrared Spectroscopy (ATR-FTIR)

ATR-FTIR spectra were recorded using a Thermo Scientific Nicolet 6700 spectrophotometer using Ge tip in the range of 4000-600 cm^{-1}

Nuclear Magnetic Resonance (NMR)

^{1}H and ^{13}C NMR experiments were recorded in a 500 MHz Bruker Avance spectrometer using deuterated solvents with internal or external TMS reference.

Solid-State Nuclear Magnetic Resonance (SS-NMR)

Solid-state NMR experiments were recorded at the Laboratoire de Chimie de Coordination (Toulouse, France) on a Bruker Avance 400 spectrometer equipped with 3.2 mm probes. Samples were spun at 10.3 and 12.5 kHz at the magic angle using ZrO_2 rotors.

Wide Angle X-ray Scattering (WAXS)

Total scattering data have been acquired on a Malvern Panalytical Empyrean III diffractometer in transmission geometry (Debye Sherrer setup). The experimental device shows the following properties: Mo source (λ(Kα)=0,7107Å); focusing mirroring; specimen in a 0.5 mm diameter capillary made of borosilicate; Galipox 3D detector. Total scattering data have been acquired on an angular range from 3 to 140 º with 0.03º angular step, for a total acquisition time of 11 h. The samples were placed with or without solvent (THF) in 0.5 mm diameter capillaries.

For the pair distribution functions (PDFs) the data was extracted using pdfgetx3 software.[1] And the corresponding PDF have been refined using the diffpy-cmi python package.[2]

Computational calculations

Periodic density functional theory (DFT) calculations were performed using the ab initio plane-wave pseudopotential approach, as implemented in the Vienna Ab initio Simulation Package (VASP). The Perdew−Burke−Ernzerhof exchange−correlation functional within the generalized gradient approximation was chosen and van der Waals interactions were taken into account through the D3 correction method of Grimme et al.[3] The supercell used was (30 × 30.5 × 31) Å large, ensuring at least 16 Å of vacuum between the successive images of Pd13. Γ-centered (1 × 1 × 1) k-point grid generated using the Monkhorst−Pack method was employed. All the systems considered are neutral. Iterative relaxation of atomic positions proceeded until the change in total energy between successive steps was less than 10^{-6} eV per cell, and the residual forces on any direction acting on the atoms (evaluated using the conjugate-gradient algorithm) were less than 0.015 eV/Å.

Catalytic experiments

Catalytic assays were carried out using 2-phenylpyridine, biphenyl, and 2,2'-bipyridine. 0.1399 mmol of substrate were used, 5 mL of THF, and 7 mol% Pd (**Pd@C$_{meso}$**), 8 mol % Pd (**Pd@PVP**), and 5 mol % Pd (**Pd@ICy, Pd@IMes**) (based on TGA analysis of the nanocatalysts), were added to a Fischer-Porter reactor. 1-4 bar of D_2 (or H_2) were added at different temperatures from 55 to 90 ºC, and at different times, ranging from 1 to 24 h. Then, an aliquot of the reaction was taken, evaporated and re-dissolved in $CDCl_3$ for NMR analysis. The conversion percentage was calculated from the relationship between the integrals from the starting substrate and product.

2. Synthesis and characterization data for imidazolium salts and free carbenes

Imidazoilum salts were synthesized according to the method described by Bantreil and Nolan for **ICyHCl** and **ICy** (**Scheme S1** and **Scheme S2**)[4].

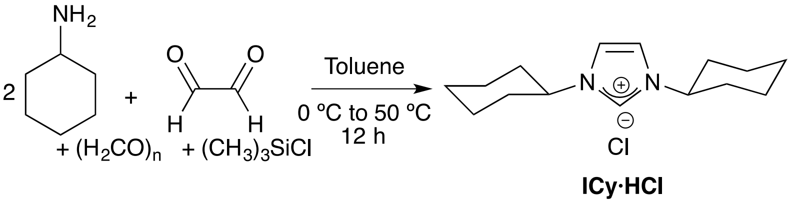

Scheme S1. Synthesis of **ICyHCl** imidazolium salt.

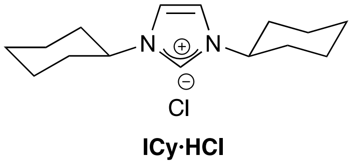

ICyHCl: cyclohexylamine (0.992 g, 10.0 mmol), toluene (100 mL) and paraformaldehyde (0.300 g, 10.0 mmol.) were added to a round-bottom flask and stirred for 30 minutes at room temperature (r.t.). The solution was cooled down at 0 ºC in an ice/water bath and a second equivalent of cyclohexylamine (0.992 g, 10.0 mmol) was added. This solution was stirred at 0 ºC for 10 minutes and then chlorotrimethylsilane (1.086 g, 1.27 mL, 10.0 mmol) was added dropwise over 40 min with vigorous stirring. The cooling bath was removed and glyoxal (40 % aqueous solution, 1.451 g, 1.150 mL, 10.0 mmol) was added. The resulting mixture was stirred for 12 hours at 50 ºC. Subsequently, the mixture was cooled at r.t. and dichloromethane and water were added to carry out the separation of the imidazolium salt. The aqueous phase was washed with dichloromethane (3x50 mL each). Finally, the solvent was evaporated, and the residue was recrystallized with isopropanol to obtain **ICyHCl** (2.20 g, 82%) (^{1}H-NMR dmso-*d*$_6$, 500 MHz): δ = 9.31 (s, 1H, CH^2 Im), 7.92 (d, $^4J_{H,H}$=1,7 Hz 2H, $CH^{4,5}$ Im), 4.29-4.23 (m, 2H, NC*H*), 2.08-2.06 (m, 4H, ICy), 1.85-1.82 (m, 4H, ICy), 1.73-1.65 (m, 6H, ICy), 1.42-1.34 (m, 4H, ICy), 1.24-1.16 (m, 2H, ICy) ppm. (**Figure S1**)

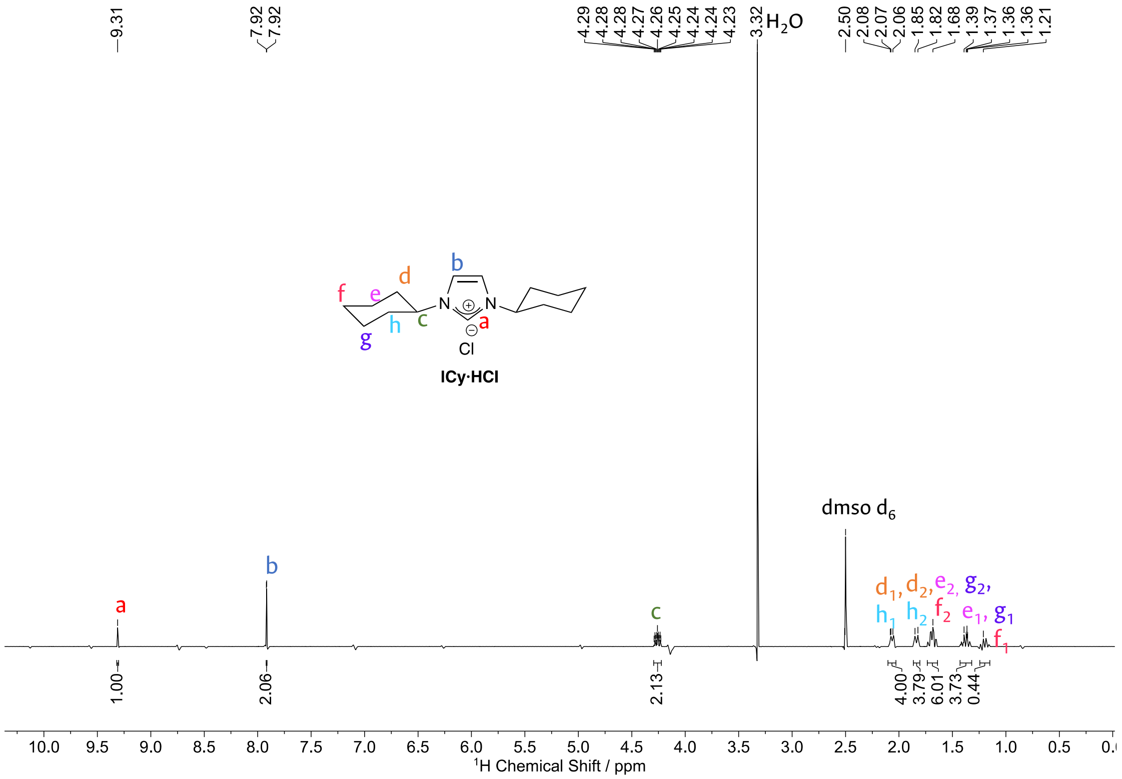

Figure S1. ^{1}H NMR (500 MHz, dmso-*d*$_6$) spectrum from **ICyHCl**.

ICy free carbene: it was synthesized before used it as stabilizer for PdNPs, and its ^{1}H NMR spectrum was recorded *in situ*. The free carbene was formed by the addition of potassium *tert*-butoxide (1.2 eq) to **ICyHCl** (1 eq) solution in THF and allowed to react for 7 hours.

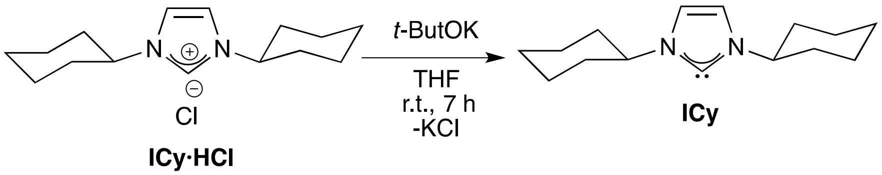

Scheme S2. Synthesis of **ICy** free carbene.

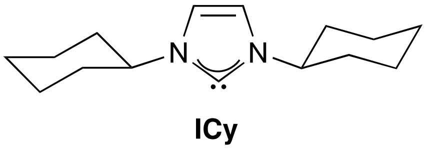

ICy: 0.0215 g (0.0799 mmol) of **ICyHCl** and 0.0108 g (0.0962 mmol) of potassium *tert*-butoxide were added in a vial with 5 mL of THF-d_8 and allowed to react over 7 h (**Scheme S2**). The NMR tube was prepared under inert atmosphere and the ^{1}H NMR spectrum was recorded immediately. (^{1}H-NMR, THF-d_8, 500 MHz): δ = 7.16 (s, 2H, $CH^{4,5}$ Im), 4.23 (s, 2H, NC*H*), 2.05-2.02 (m, 4H, ICy), 1.85-1.82 (m, 4H, ICy), 1.79-1.67 (m, 6H, ICy), 1.48-1.40 (m, 4H, ICy), 1.31-1.26 (m, 2H, ICy) ppm. (**Figure S2**)

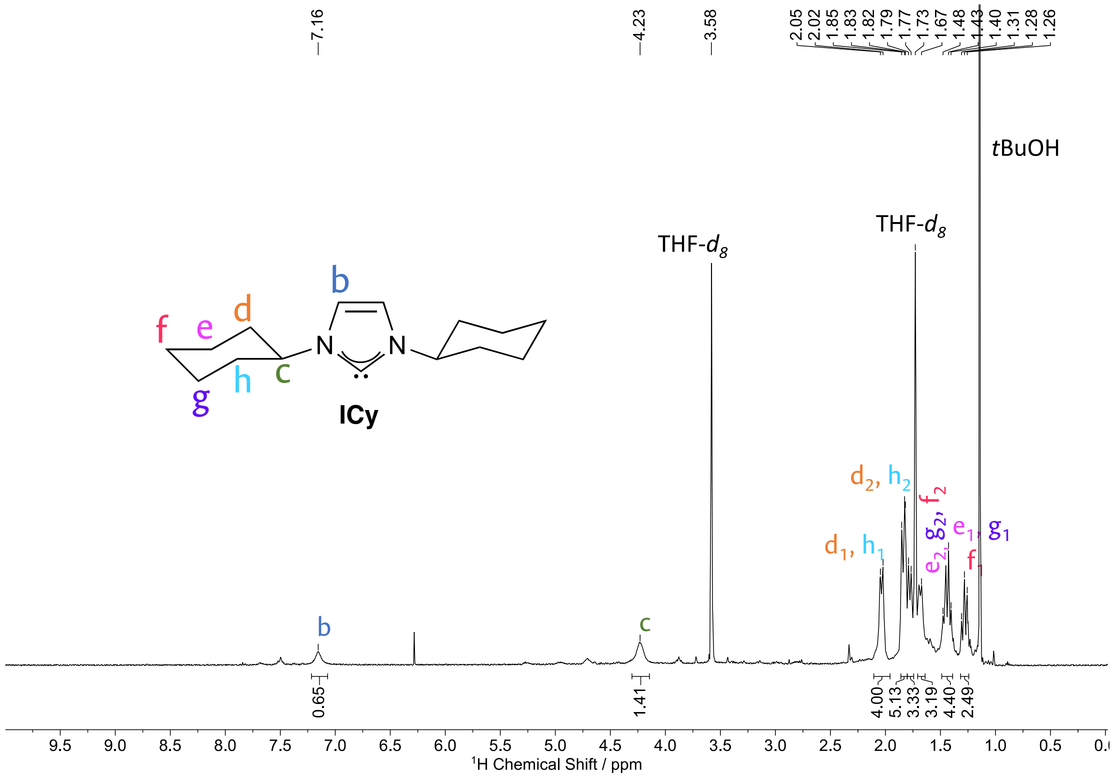

Figure S2. ^{1}H NMR spectrum (500 MHz, THF-d_8) of **ICy** free carbene.

3. Synthesis and characterization data for Pd nanoparticles

All the synthesis of the nanoparticles were carried out inside the glove box and under inert atmosphere. Palladium nanoparticles were synthesized using the procedure designed by Chaudret and co-workers known as organometallic approach.[5]

Pd nanoparticles were synthesized by the addition of the stabilizer (**PVP** or **ICy**).

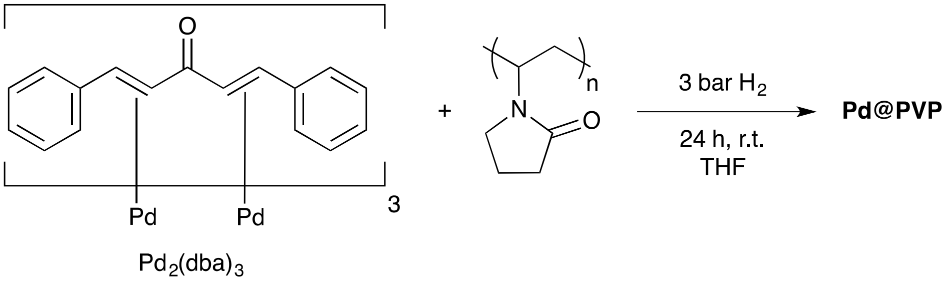

Scheme S3. Synthesis of Pd nanoparticles stabilized with PVP (**Pd@PVP**).

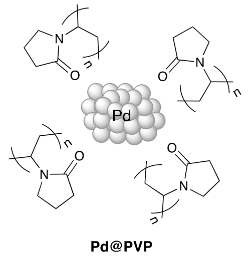

Pd@PVP: Pd nanoparticles stabilized with PVP were synthesized according to the method described by Pieters and co-workers (**Scheme S3**).[6] 109.5 mg (0.1196 mmol) of $Pd_2(dba)_3$ were added to a Fischer-Porter with THF (30 mL) under inert atmosphere. Subsequently, 0.475 g of PVP were dissolved in THF (30 mL) and added to the Pd solution. Then, 3 bar of H_2 were added and allowed to react at rt overnight. Finally, the NPs were precipitated and washed with pentane (3 x 30 mL) and dried under vacuum overnight (92 % based on Pd according to TGA analysis). **TEM:** 1.9 ± 0.2 nm.

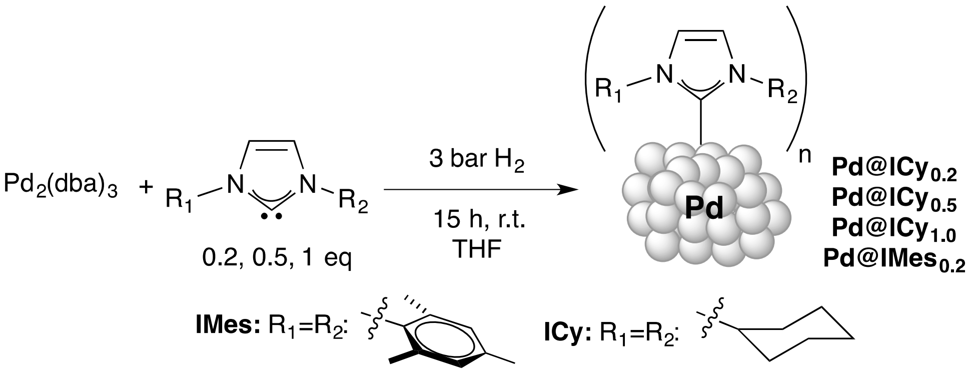

Scheme S4. Pd nanoparticles synthesis stabilized by **NHC ligands** (**Pd@ICy**).

Pd@ICy: The free carbene was formed by the addition of potassium *tert*-butoxide (1.2 eq) to the imidazolium salt (1 eq) solution in THF and allowed to react for 7 hours. This solution was filtered through Celite® to remove the salt excess. After, three different systems were synthesized, depending on the Pd:NHC ratio (**Scheme S4**). Thus, 0.183 g (0.1998 mmol) of $Pd_2(dba)_3$ were added to a Fischer-Porter with THF (15 mL) under inert atmosphere. Subsequently, the filtered free carbene solution was added (0.2, 0.5 and 1 eq). To this solution, 3 bar of H_2 were added and allowed to react at r.t. overnight. Finally, the PdNPs were precipitated and washed with pentane (3 x 30 mL) and dried under vacuum overnight.

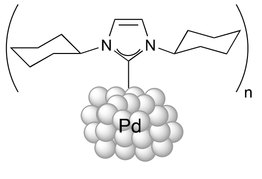

Pd@ICy$_{0.2}$

Pd@ICy$_{0.2}$: 0.0215 g (0.0799 mmol) of **ICyHCl** and 0.0108 g (0.0962 mmol) of potassium *tert*-butoxide were used, along with 0.183 g (0.1998 mmol) of $Pd_2(dba)_3$ following the procedure above mentioned (69.1 % yield based on Pd according to TGA analysis). **Pd:NHC ratio:** 5:1. **TEM:** 1.9 ± 0.2 nm. **HRTEM**: fcc Pd nanoparticles.

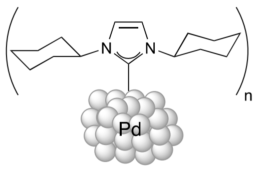

Pd@ICy$_{0.5}$

Pd@ICy$_{0.5}$: 0.0546 g (0.203 mmol) of **ICyHCl** and 0.0279 g (0.249 mmol) of potassium *tert*-butoxide were used, along with 0.183 g (0.1998 mmol) of $Pd_2(dba)_3$ following the procedure above mentioned (49.5 % yield based on Pd according to TGA analysis). **Pd:NHC ratio:** 2:1. **TEM:** 1.7 ± 0.2 nm.

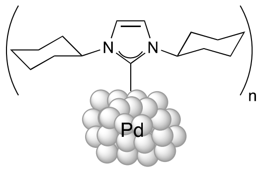

Pd@ICy$_{1.0}$

Pd@ICy$_{1.0}$: 0.1075 g (0.399 mmol) of **ICyHCl** and 0.0534 g (0.476 mmol) of potassium *tert*-butoxide were used, along with 0.183 g (0.1998 mmol) of $Pd_2(dba)_3$ following the procedure above mentioned. No NPs were obtained in this case.

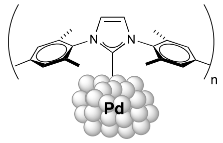

Pd@IMes: 0.183 g (0.1998 mmol) of $Pd_2(dba)_3$ were added to a Fischer-Porter with THF (15 mL) under inert atmosphere. Subsequently 0.024 g (0.0799 mmol) of **IMes** with THF (15 mL) were added to the reactor. To this solution, 3 bar of H_2 were added and allowed to react at r.t. overnight. Finally, the PdNPs were precipitated and washed with pentane (3 x 30 mL) and dried under vacuum overnight. (65.4 % yield based on Pd according to TGA analysis). **TEM:** 1.8 ± 0.3 nm

$$Pd_2(dba)_3 + \text{Mesoporous carbon} \xrightarrow[\text{24 h, r.t., THF}]{\text{3 bar } H_2} \mathbf{Pd@C_{meso}}$$

Scheme S5. Synthesis of Pd nanoparticles supported on mesoporous carbon (**Pd@C$_{meso}$**).

Pd@C$_{meso}$ (Scheme S5): 215.0 mg (0.2349 mmol) of $Pd_2(dba)_3$ were added to a Fischer-Porter under inert atmosphere. Then, 450 mg of mesoporous carbon was added to the flask with THF (5 mL). The solution was stirred during 24 hours at r.t. Then, 3 bar of H_2 were added to the solution and allowed to react during 24 hours at r.t. Finally, the NPs were precipitated and washed with pentane (3 x 30 mL) and dried under vacuum overnight (86 % based on Pd). **TEM:** 2.6 ± 0.4 nm.

4. XRD measurements for PdNPs

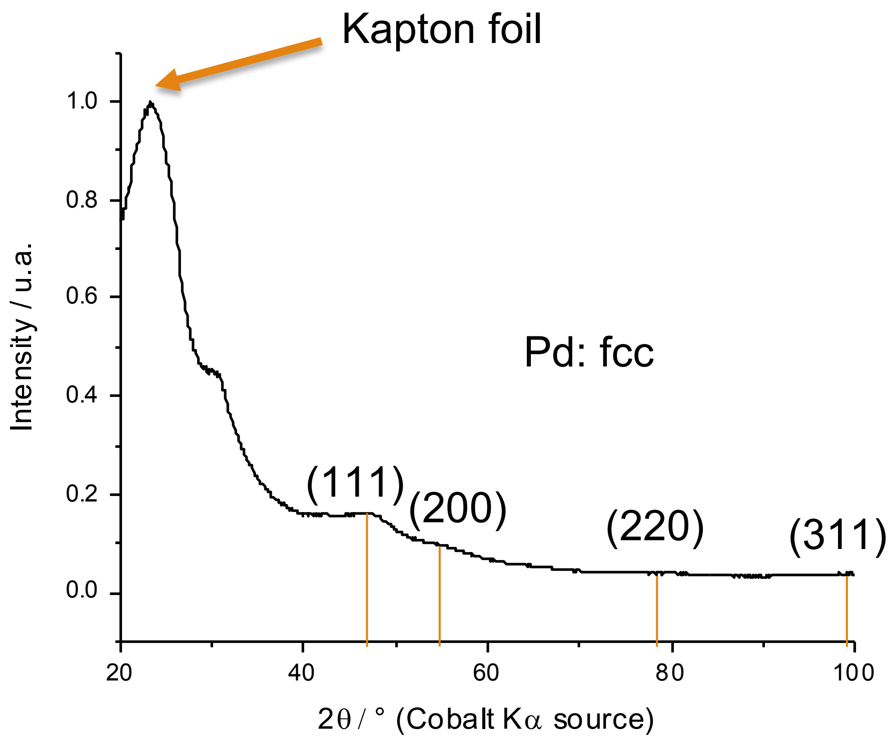

Figure S3. XRD diffractogram for **Pd@PVP** NPs.

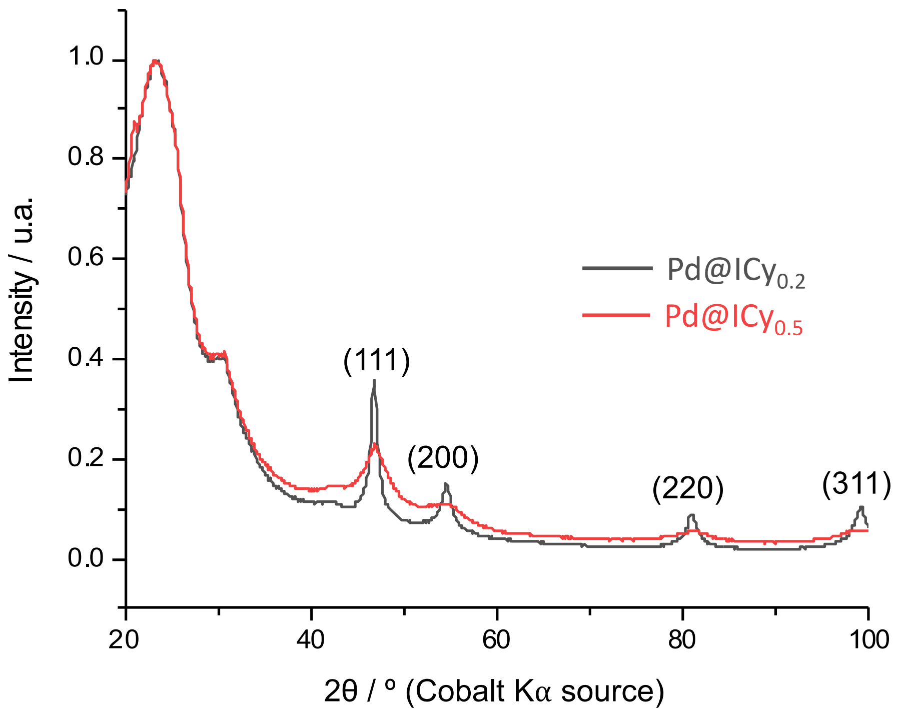

Figure S4. XRD diffractogram for **Pd@ICy** systems.

5. WAXS for PdNPs

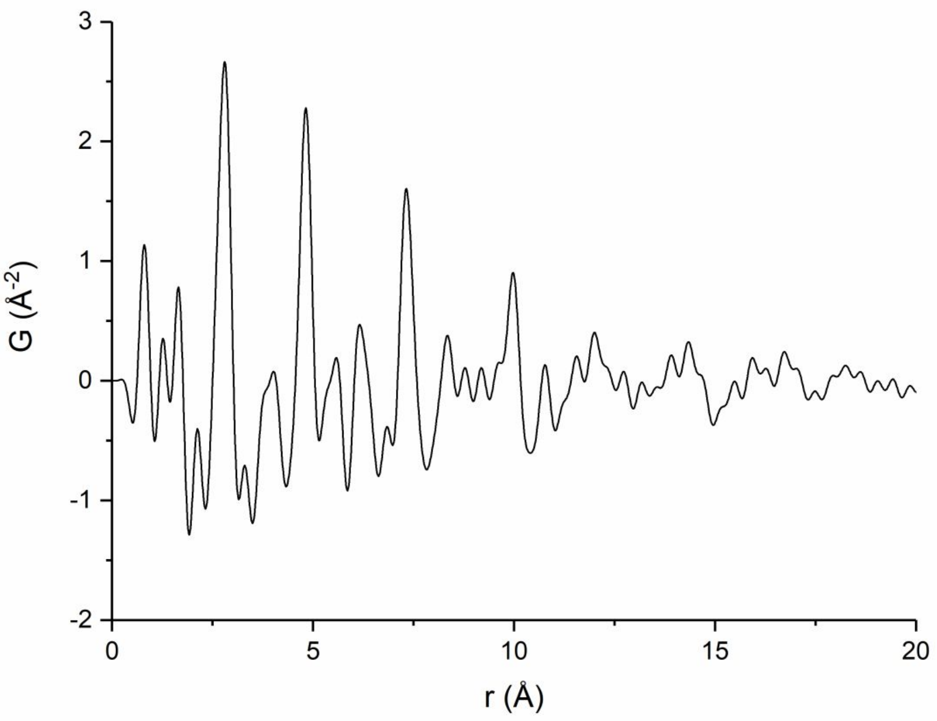

Figure S5. Pair Distribution Function of **Pd@IMes$_{0.2}$** NPs.

6. TEM images for PdNPs

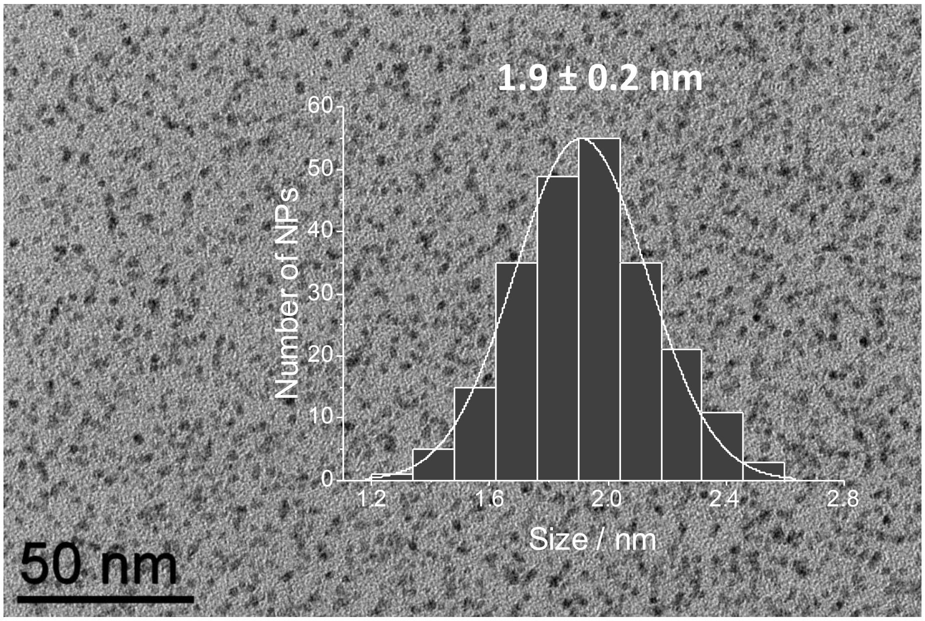

Figure S6. TEM micrograph and size distribution for **Pd@PVP** NPs.

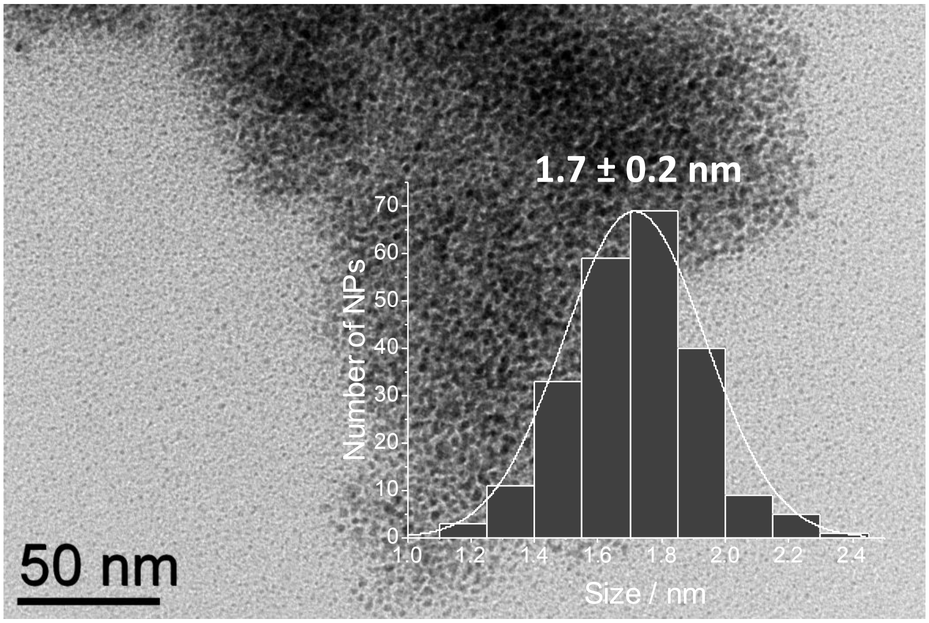

Figure S7. TEM micrograph and size distribution for **Pd@ICy$_{0.5}$** NPs.

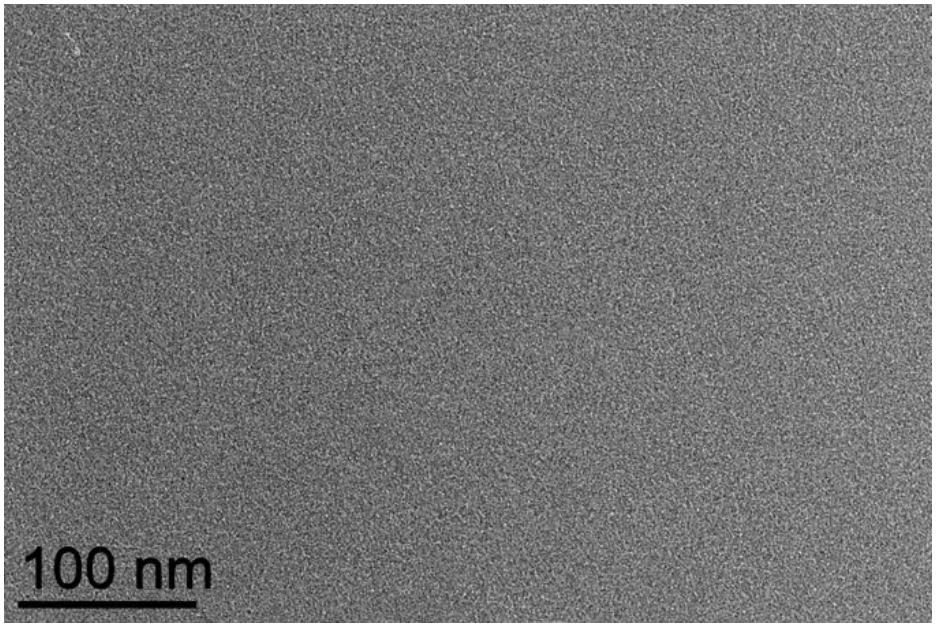

Figure S8. TEM micrograph for **Pd@ICy$_{1.0}$**. No NPs were observed in this sample.

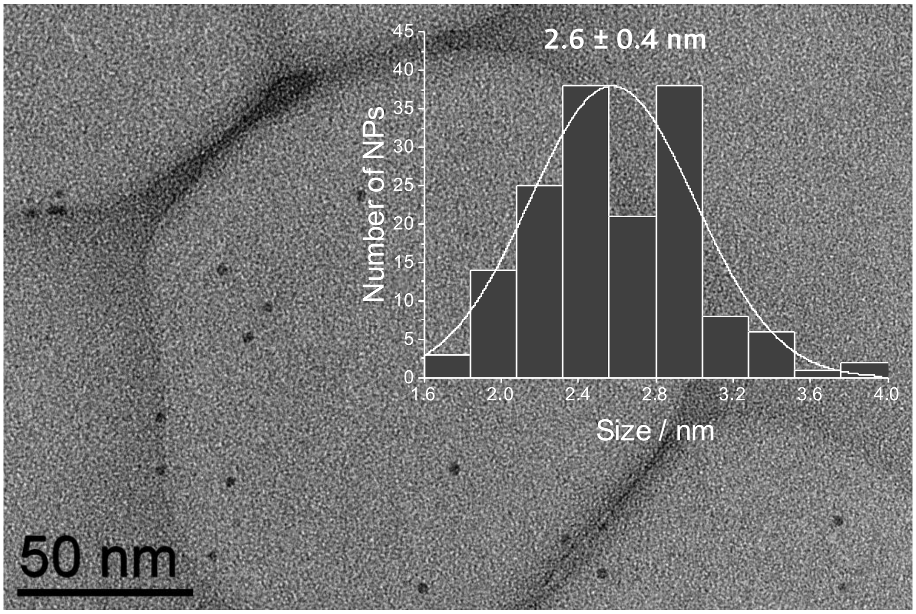

Figure S9. TEM micrograph and size distribution for **Pd@C$_{meso}$** NPs.

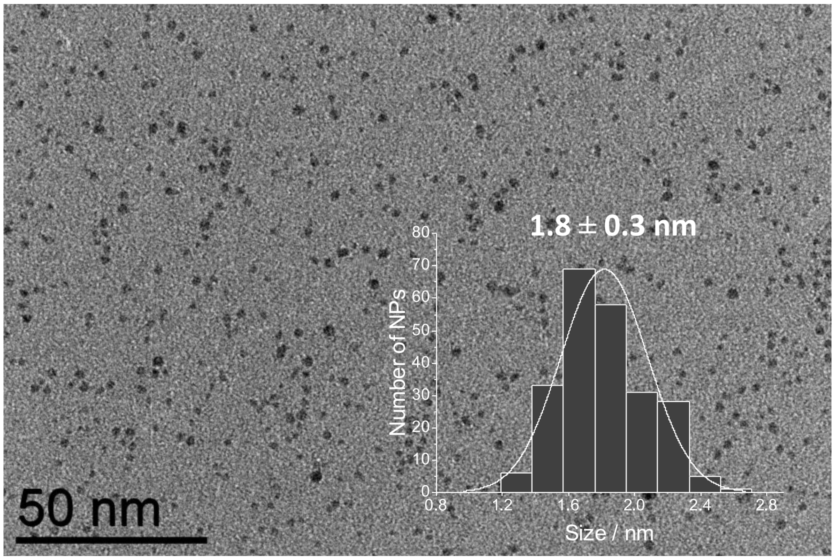

Figure S10. TEM micrograph and size distribution for **Pd@IMes$_{0.2}$** NPs.

Table S1. Sizes from the synthesized PdNPs

Entry	PdNPs	Size (nm)
1	**Pd@PVP**	1.9 ± 0.2
2	**Pd@ICy$_{0.2}$**	1.9 ± 0.2
3	**Pd@ICy$_{0.5}$**	1.7 ± 0.2
4	**Pd@C$_{meso}$**	2.6 ± 0.4
5	**Pd@IMes$_{0.2}$**	1.8 ± 0.3

7. EDX spectra for PdNPs

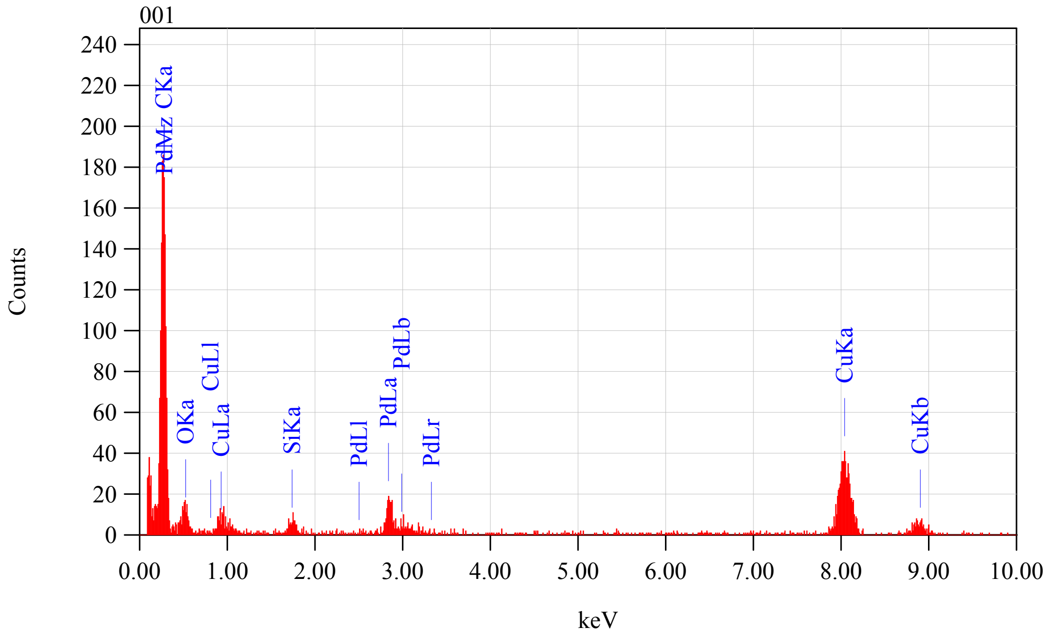

Figure S11. EDX spectrum for **Pd@ICy$_{0.2}$** NPs.

8. ATR-FTIR spectra for PdNPs

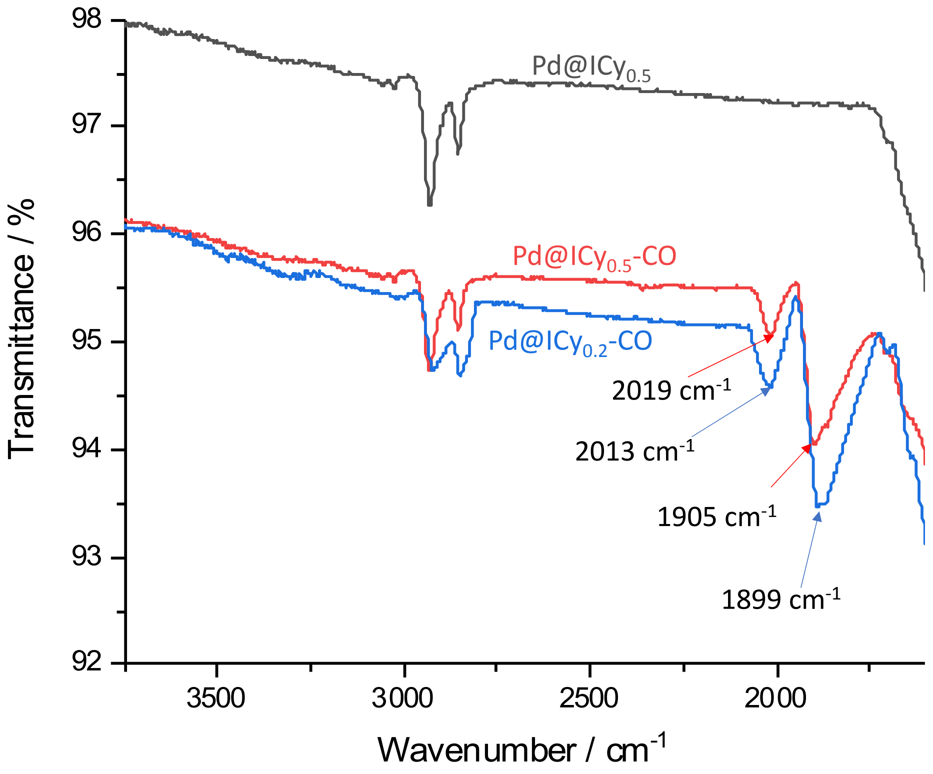

Figure S12. ATR-FTIR spectrum for **Pd@ICy$_{0.5}$** before exposure with CO (gray), **Pd@ICy$_{0.5}$** (red) and **Pd@ICy$_{0.2}$** (blue) after exposure with CO.

In order to gain insights into the surface state of the NPs, we exposed **Pd@ICy** NPs with 1 bar of CO overnight and recorded the ATR-FTIR spectra. As shown on Figure S13, we can determine that both **Pd@ICy$_{0.2}$** and **Pd@ICy$_{0.5}$** NPs have CO coordinated in bridging (CO_b) and terminal (CO_t) mode at ~ 1900 cm^{-1} and ~ 2020 cm^{-1}, respectively. It suggests that NHCs are mainly located at the edges, and/or corners leaving the faces available for CO coordination. Furthermore, as expected, in the system **Pd@ICy$_{0.2}$** the CO_b signal has greater intensity compared with the **Pd@ICy$_{0.5}$** NPs. It clearly shows that the faces are more available for the substrate coordination in the former compared to the latter.[7]

9. Solid-State ^{1}H–^{13}C CP-MAS NMR spectra for PdNPs

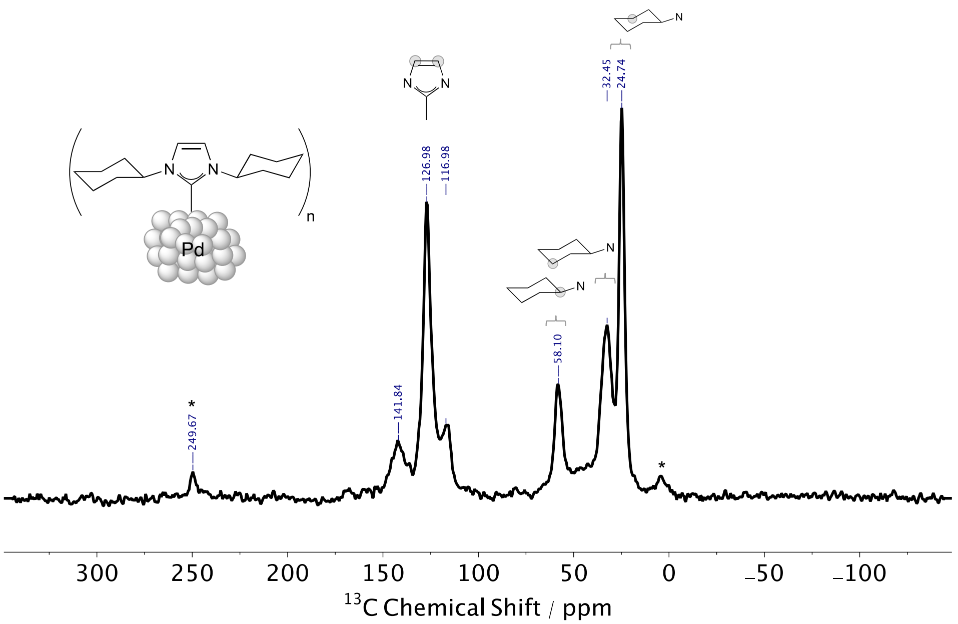

Figure S13. Solid-State ^{1}H–^{13}C CP-MAS NMR (100 MHz, spin at 12.5 kHz) for **Pd@ICy$_{0.2}$**. (*: side spinning band)

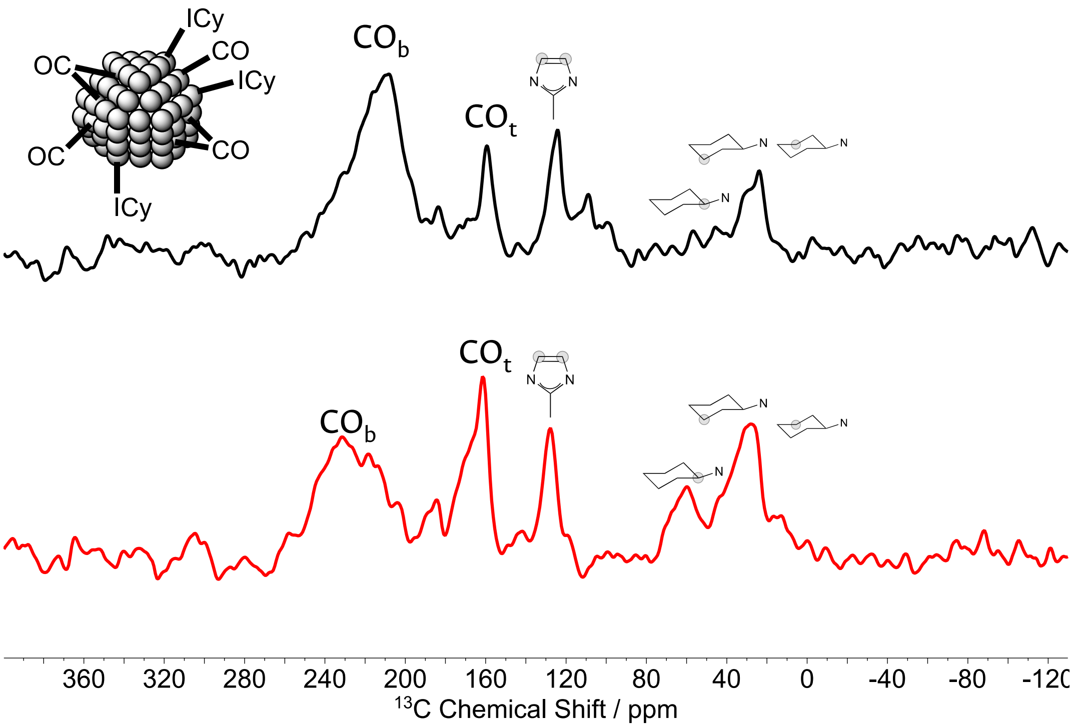

Figure S14. Solid-State ^{13}C MAS (black) and ^{1}H–^{13}C CP-MAS (red) NMR (100 MHz, spin at 12.5 kHz) spectra for **Pd@ICy$_{0.2}$** after exposure with ^{13}CO.

A way to characterize the surface state of the PdNPs is Solid-State NMR (SS-NMR). Thus, we recorded the ^{1}H–^{13}C Cross Polarization Magic Angle Spinning (CP-MAS) SS-NMR spectrum for the **Pd@ICy$_{0.2}$** NPs. We observed resonance signals at 126 and 58-32 ppm attributed to the imidazole ring and the cyclohexyl moieties, respectively. The resonance signal of the carbenic carbon (expected at 170−190 ppm for NPs of similar sizes displaying no Knight shift)[8] was not observed in the spectrum, which is, however, consistent with the literature due to significant peak broadening effects close to the surface (Figure S14).[7,9–12] Nevertheless, it is worth to mention that no signal was detected at the protonated position (imidazolium salt, 155 ppm), suggesting that the NHC is coordinated to the metal surface. Thus, the coordination of the carbenic carbon to the PdNPs surface explains the high colloidal stability observed for these systems.[9]

Additionally, we exposed the **Pd@ICy$_{0.2}$** NPs with 1 bar of ^{13}CO overnight and recorded the ^{13}C MAS and ^{1}H–^{13}C CP-MAS SS-NMR spectra (Figure S15). As it can be observed, two new resonance signals appeared at 160 and 220 ppm corresponding to $^{13}CO_t$ and $^{13}CO_b$, respectively.[10] It should be noted that, the $^{13}CO_b$ signal has higher intensity in the ^{13}C MAS compared to the ^{1}H–^{13}C CP-MAS spectrum. This is due to the high mobility of the $^{13}CO_b$ suggesting that the faces of the NPs are totally available for coordination of, for example, substrates which is crucial for catalysis. In contrast, the $^{13}CO_t$ resonance signal is enhanced in the CP-MAS experiment indicating a restricted mobility for these ligands on the surface. Thus, it allows us to deduce that the NHC ligands are coordinated at the edges of the NPs avoiding the mobility of the $^{13}CO_t$ coordinated preferentially at the corners of the NP.[13,14]

10. NMR and Mass spectra for deuteration reactions

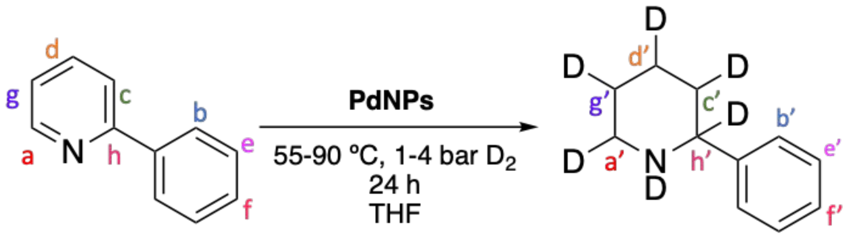

Scheme S6. Reduction of 2-phenylpyridine to 2-phenylpiperidine-d_6 using PdNPs.

a)

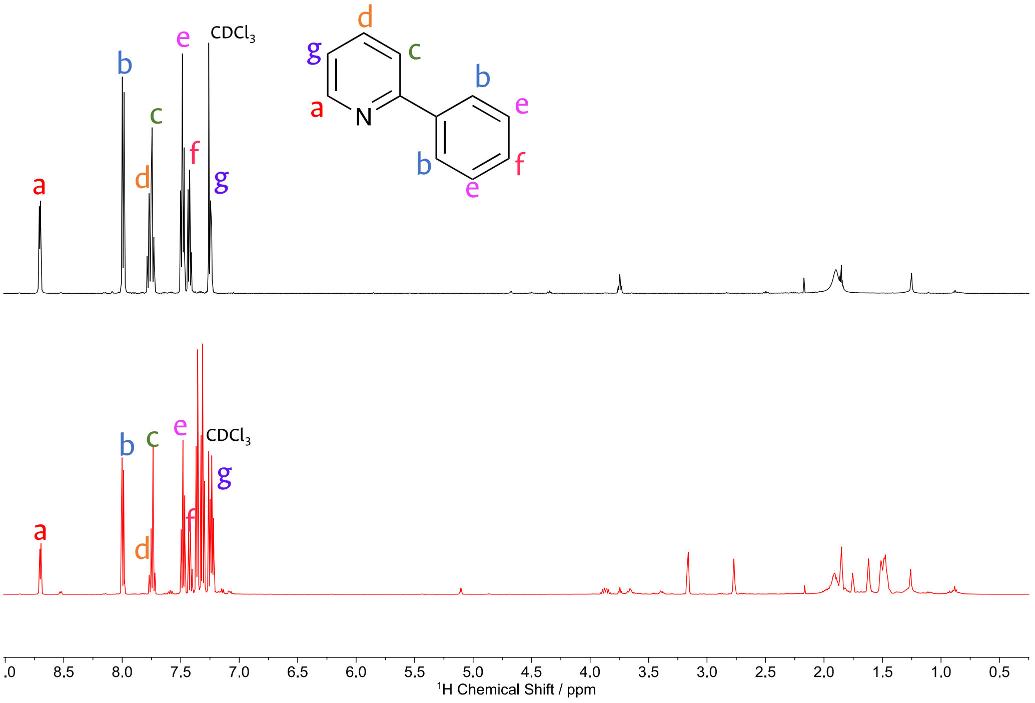

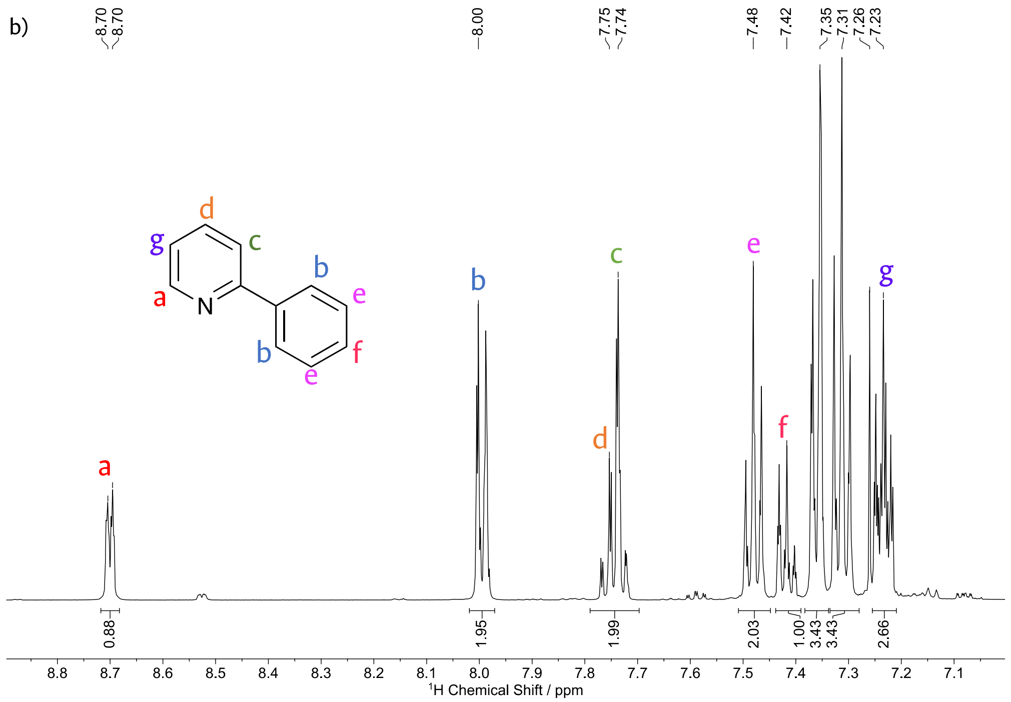

Figure S15. a) ^{1}H NMR spectrum (500 MHz, $CDCl_3$) for 2-phenylpyridine (black) and 2-phenylpyridine dearomatization (red) using **Pd@ICy$_{0.2}$** (55 ºC, 24 h, 4 bar D_2, 5 mol % Pd), b) integral values for 2-phenylpyridine dearomatization with the same conditions previously mentioned.

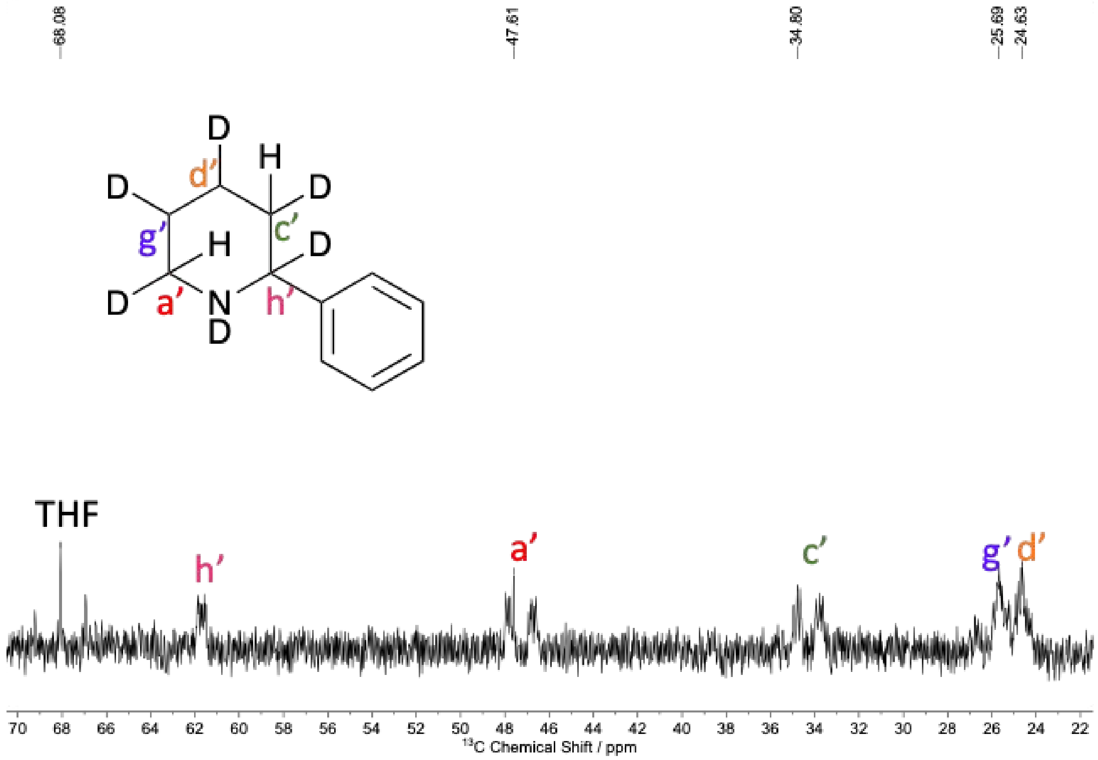

Figure S16. ^{13}C (coupled to ^{1}H) NMR spectrum (125 MHz, $CDCl_3$) for 2-phenylpyridine dearomatization.

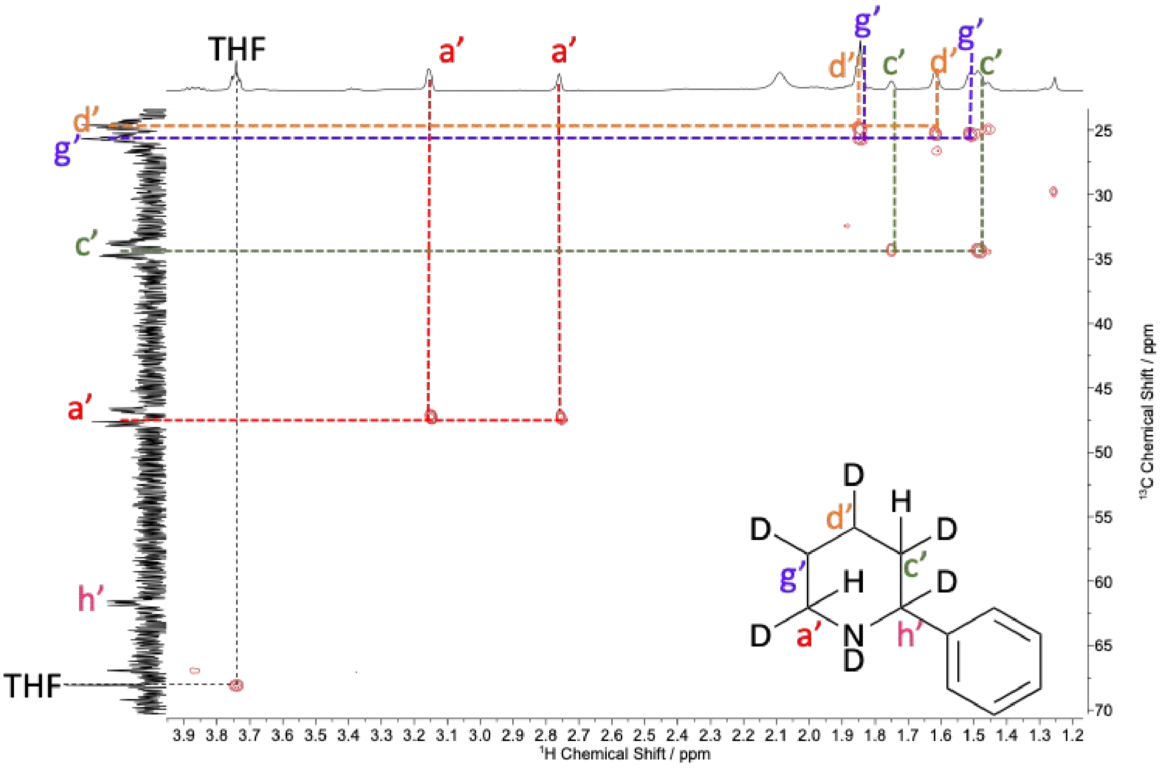

Figure S17. $^1H–^{13}C$ HSQC-NMR ($CDCl_3$) spectrum for 2-phenylpyridine dearomatization.

2-phenylpiperidine-d_6 NMR elucitation.

The appearance of new aromatic and alkyl resonance signals at 7.3 and 3.2–1.4 ppm, respectively, in the ^1H NMR spectrum (Figure S15) attributed to 2-phenylpiperidine-d_6 (Scheme 1), suggesting that a reduction reaction took place selectively at the heterocycle. To confirm the structure, we recorded the ^{13}C (coupled to ^1H) and $^1H–^{13}C$ HSQC NMR spectra (Figures S16 and S17). On the ^{13}C NMR spectrum (Figure S16), we can see different new signals in the aliphatic zone. The most remarkable peak is the one at 62 ppm, which belongs to the carbon of the piperidyl ring linked to the phenyl group (h’ in the spectrum, Figure S16). It is highly deshielded due to its proximity to both an aromatic ring and a nitrogen atom. Importantly, this signal is a triplet with 1:1:1 intensity due to the $^{13}C–^2H$ coupling. In addition, we observed the signals for the a’ and c’ carbons at 47 and 37 ppm, respectively. Their multiplicity was found to be doublets of triplets, caused by both $^{13}C–^1H$ and $^{13}C–^2H$ coupling. Moreover, the absence of any cross peak in the signal of the h’ carbon on the $^1H–^{13}C$ HSQC-NMR spectrum (Figure S17) confirmed that the 2-phenylpiperidine-d_6 was the product. Additionally, this experiment revealed the presence of a mixture of stereoisomers of the 2-phenylpiperidine-d_6 evidenced by the coupling between one carbon atom and two different protons (denoted as a’, c’, d’ and g’ in the Figure S17) depending on their axial or equatorial position. Mass Spectrum of the sample supports the formation of the product (Figure S18, molecular ion observed: m/z = 167.08 uma, expected at m/z = 167.2 uma).

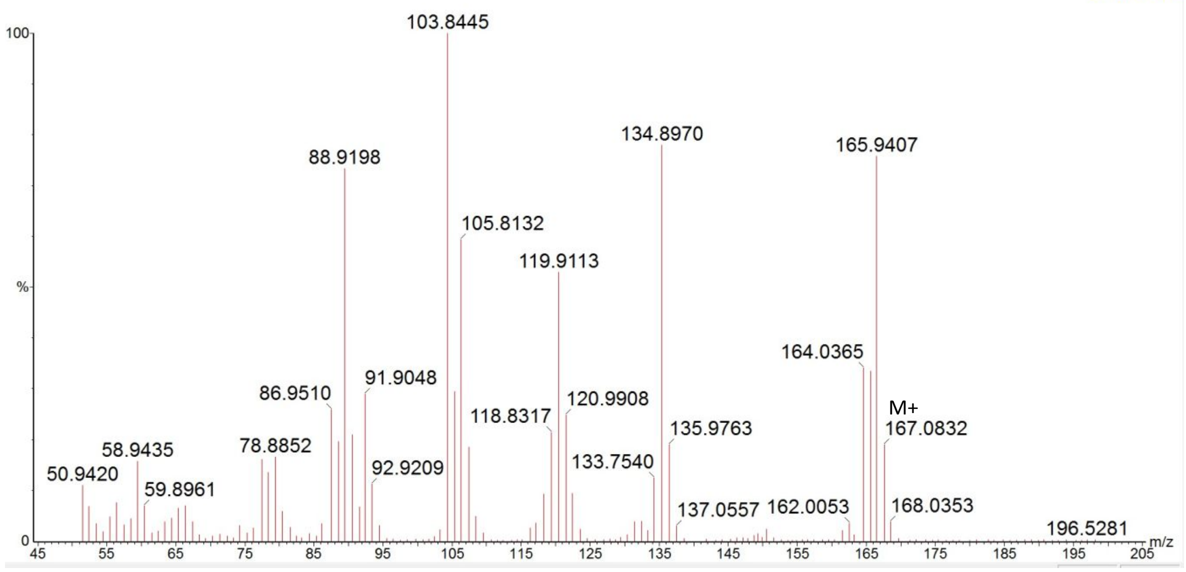

Figure S18. Mass spectrum for 2-phenylpyperidine-d_6 obtained in the dearomatization reaction catalyzed by **Pd@ICy$_{0.2}$** (55 ºC, 24 h, 4 bar D_2, 5 mol % Pd).

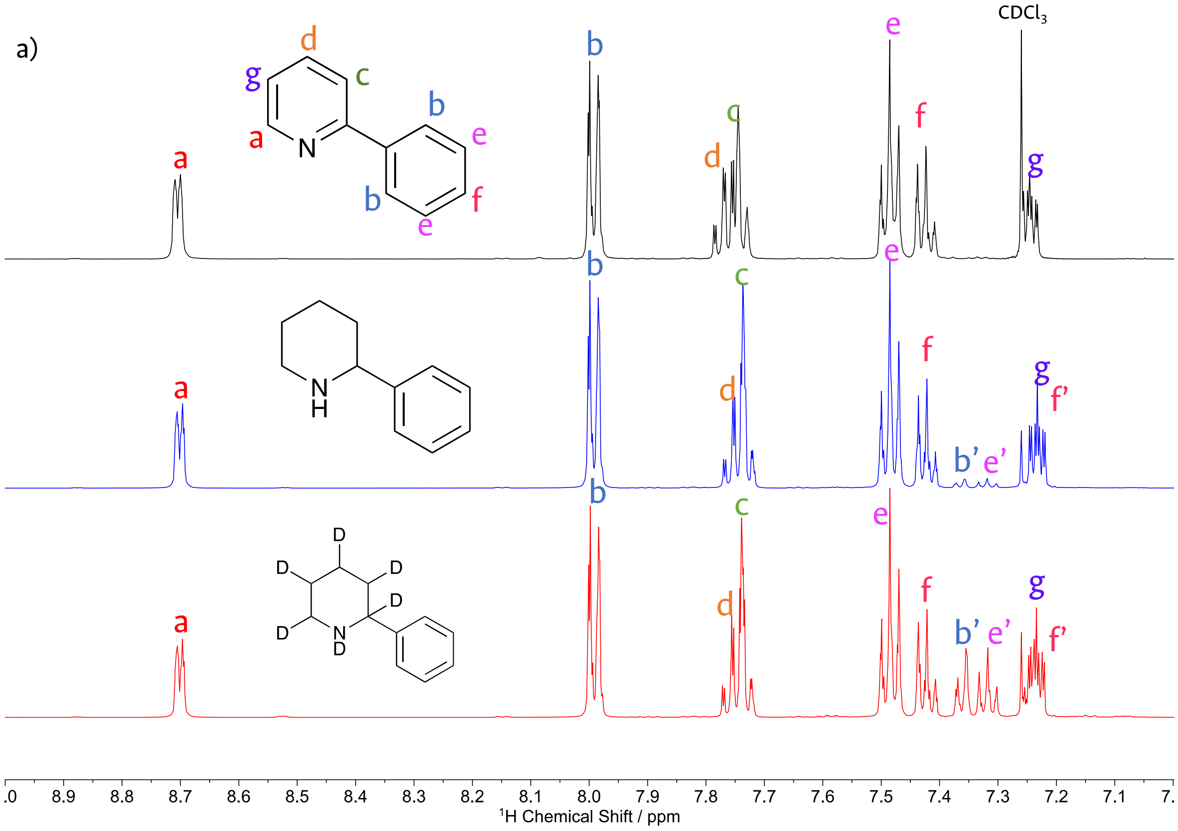

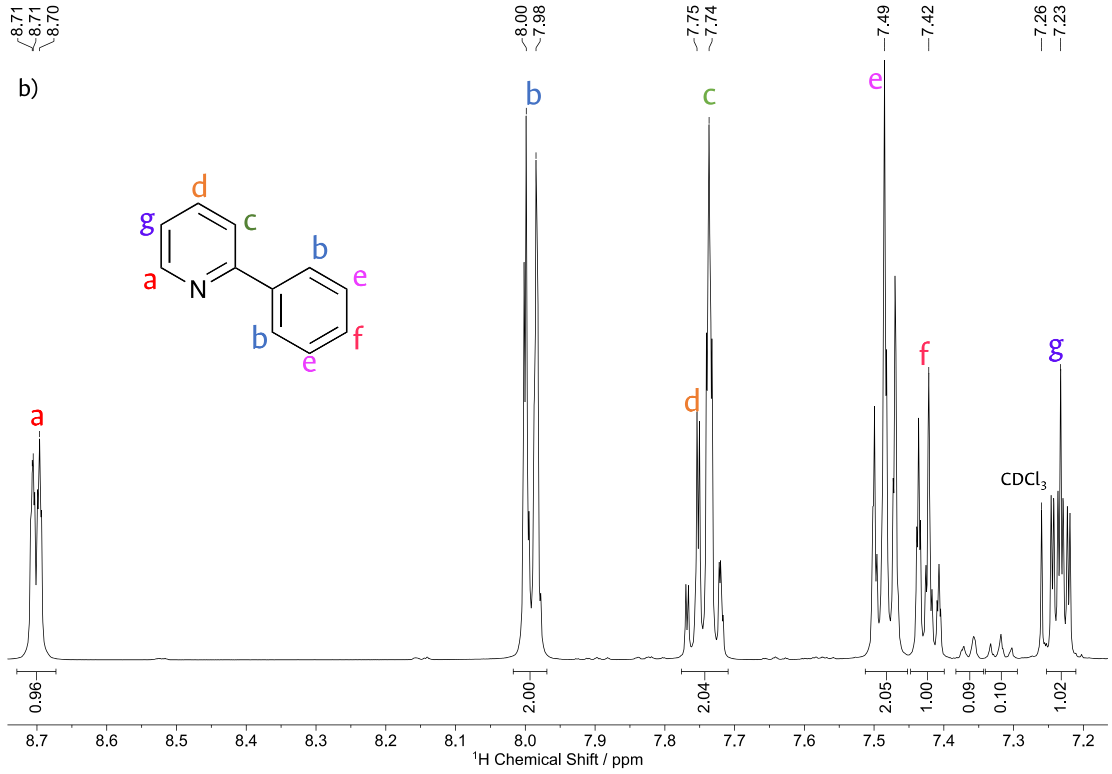

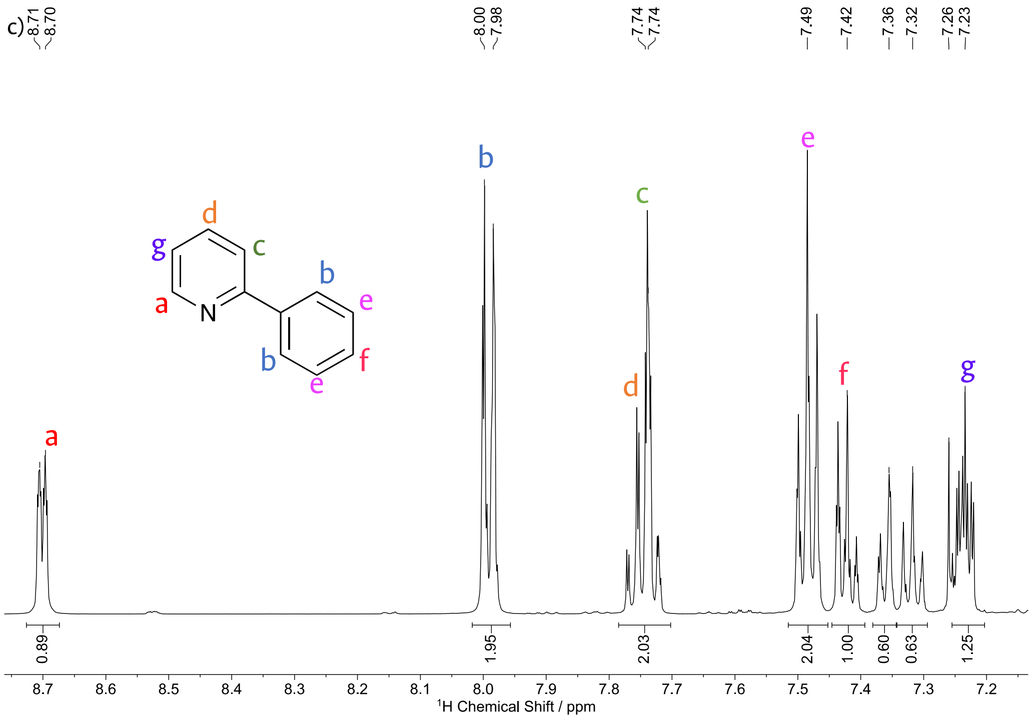

Figure S19. a) ^{1}H NMR spectrum (500 MHz, $CDCl_3$) for 2-phenylpyridine (black) and 2-phenylpyridine dearomatization using **Pd@ICy$_{0.2}$** (24 h, 70 ºC, 4 bar H_2, 5 mol % Pd) (blue) and **Pd@ICy$_{0.2}$** (24 h, 70 ºC, 4 bar D_2, 5 mol % Pd) (red), b) integral values for 2-phenylpyridine dearomatization with the same conditions previously mentioned using H_2, c) integral values for 2-phenylpyridine dearomatization with the same conditions previously mentioned using D_2.

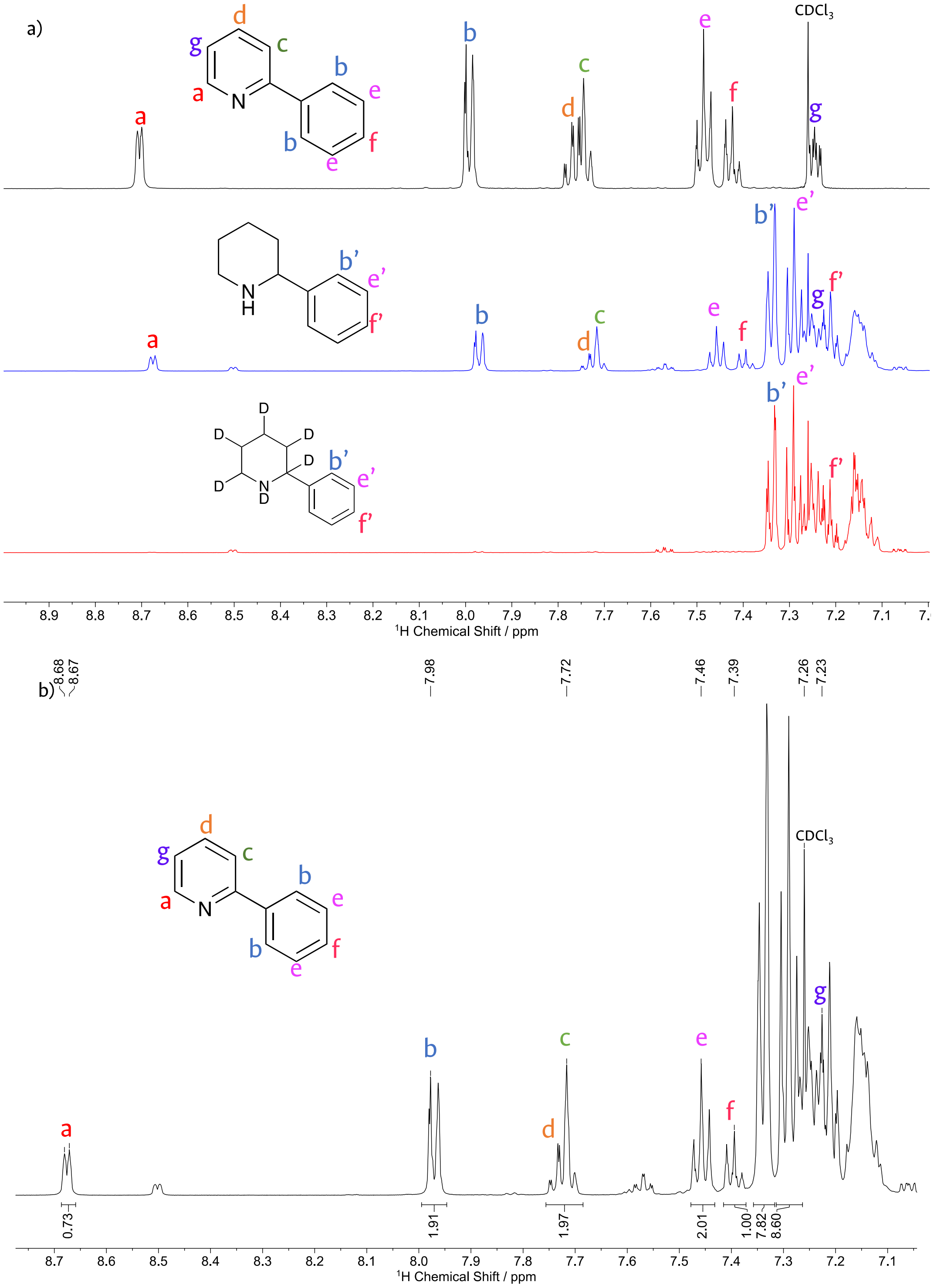

Figure S20. a) ^{1}H NMR spectrum (500 MHz, $CDCl_3$) for 2-phenylpyridine (black) and 2-phenylpyridine dearomatization using **Pd@PVP** (24 h, 70 ºC, 4 bar H_2, 8 mol % Pd) (blue) and **Pd@PVP** (24 h, 70 ºC, 4 bar D_2, 8 mol % Pd) (red), b) integral values for 2-phenylpyridine dearomatization with the same conditions previously mentioned using H_2.

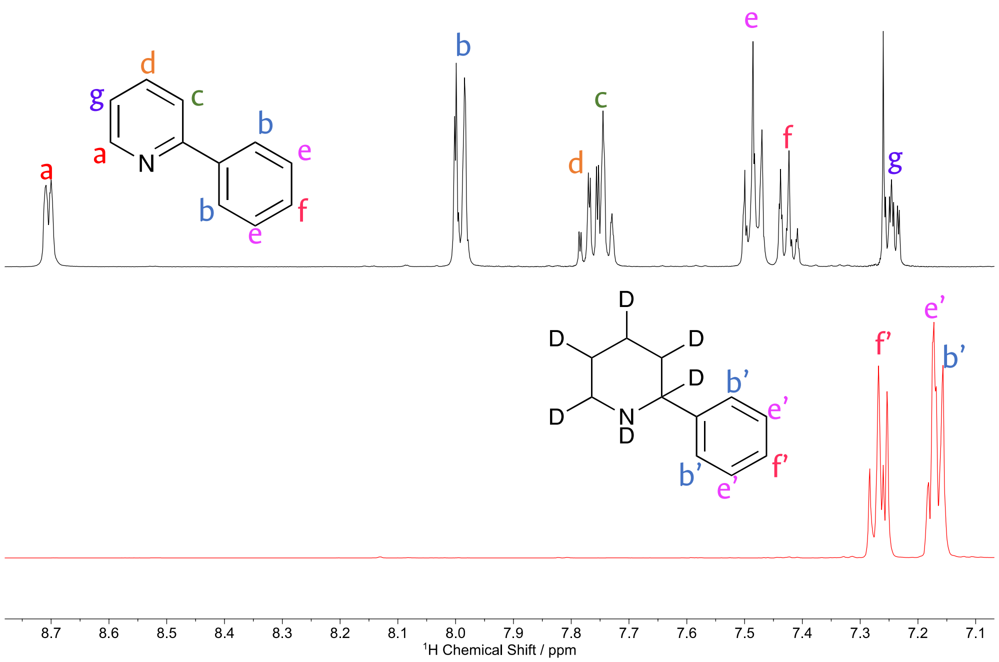

Figure S21. ^{1}H NMR spectrum (500 MHz, $CDCl_3$) for 2-phenylpyridine (black) and 2-phenylpyridine dearomatization (red) using **Pd@C$_{meso}$** (70 ºC, 12 h, 4 bar D_2, 7 mol % Pd).

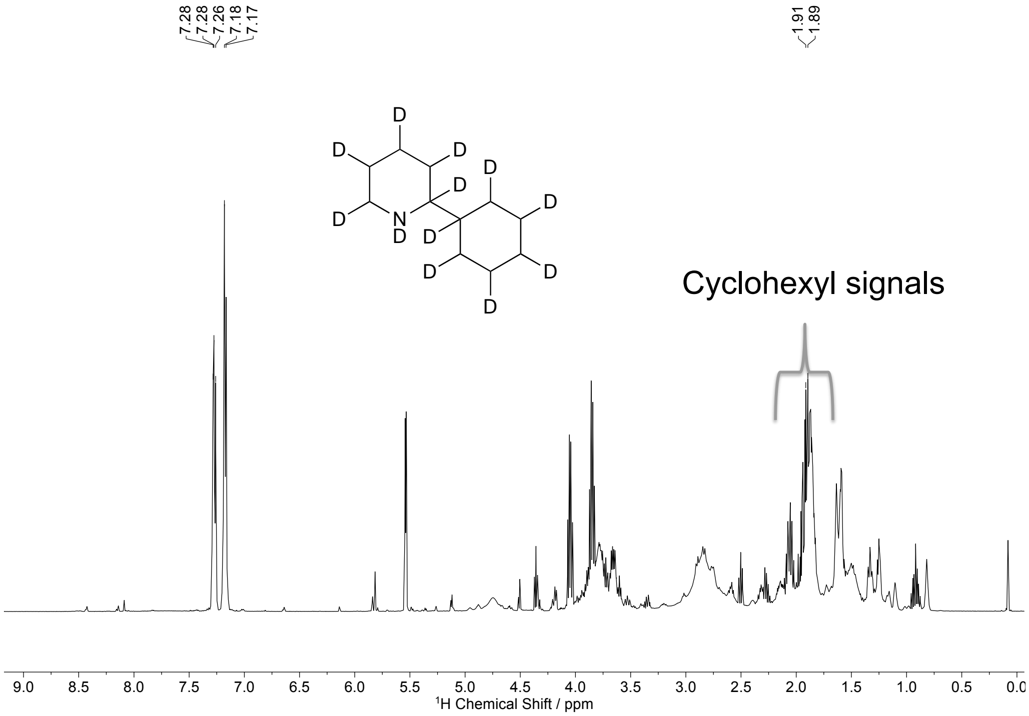

Figure S22. ^{1}H NMR spectrum (500 MHz, $CDCl_3$) for 2-phenylpyridine dearomatization using **Pd@C$_{meso}$** (90 ºC, 12 h, 4 bar D_2, 7 mol % Pd).

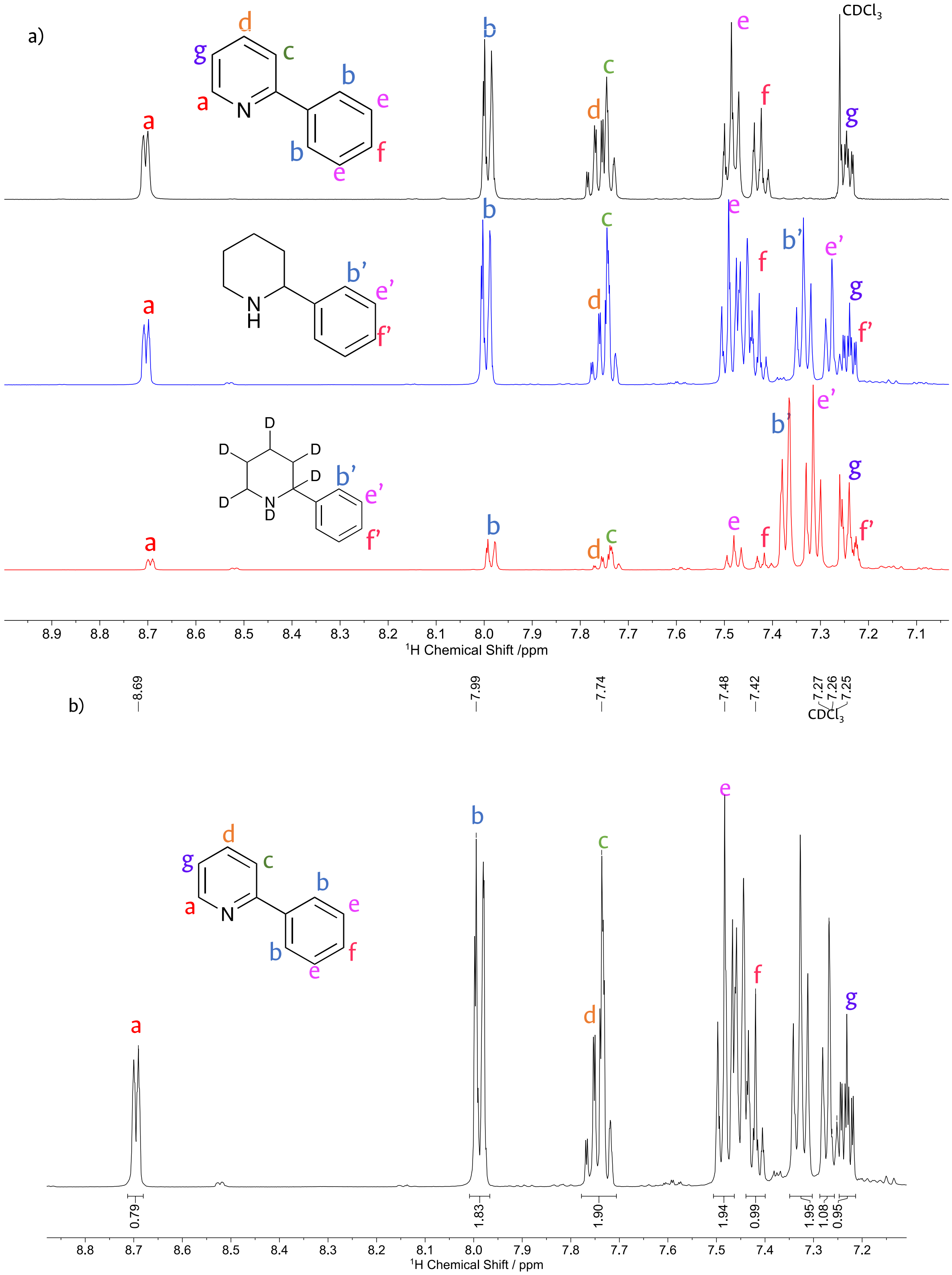
a)
CDCl3
^{1}H Chemical Shift /ppm
b)
8.69
7.99
7.74
7.48
7.42
7.27
7.26
7.25
CDCl3
0.79
1.83
1.90
1.94
0.99
1.95
1.08
0.95
^{1}H Chemical Shift / ppm

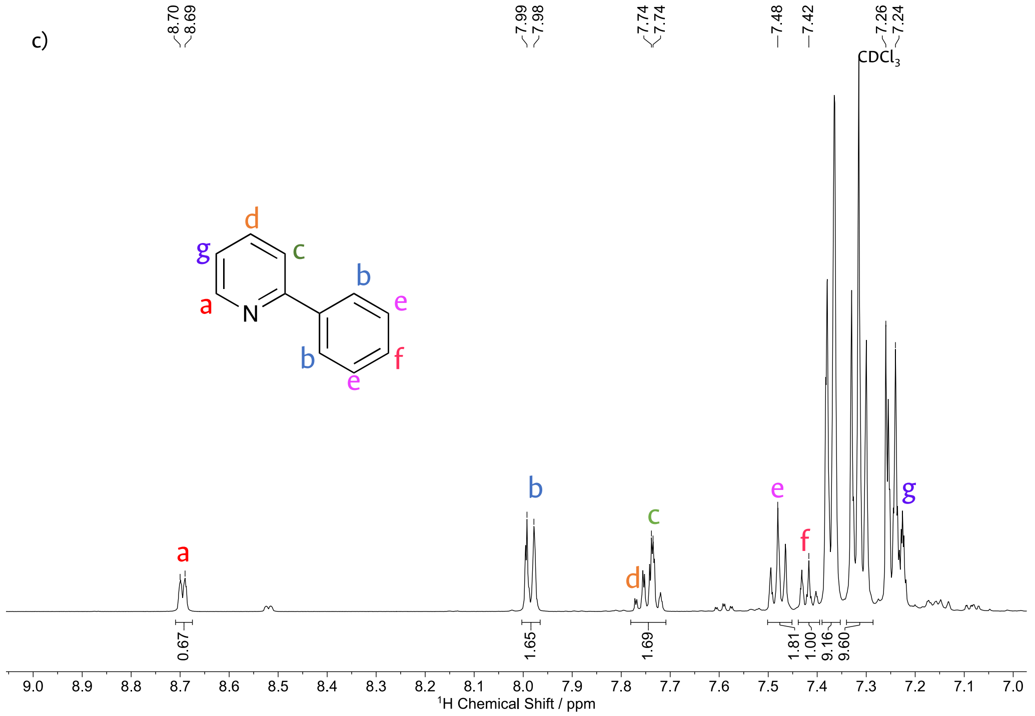

Figure S23. a) ^{1}H NMR spectrum (500 MHz, $CDCl_3$) for 2-phenylpyridine (black) and 2-phenylpyridine dearomatization using **Pd@IMes$_{0.2}$** (24 h, 55 ºC, 2 bar H_2, 5 mol % Pd) (blue) and **Pd@IMes$_{0.2}$** (24 h, 55 ºC, 2 bar D_2, 5 mol % Pd) (red), b) integral values for 2-phenylpyridine dearomatization with the same conditions previously mentioned using H_2, c) integral values for 2-phenylpyridine dearomatization with the same conditions previously mentioned using D_2.

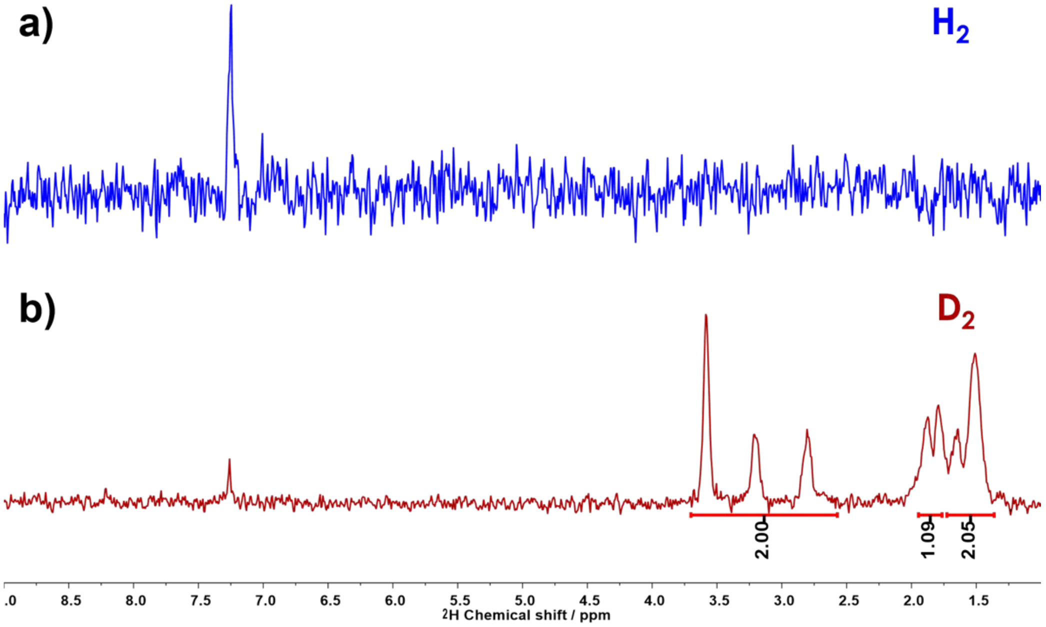

Figure S24. ^{2}H NMR spectrum (61 MHz, $CHCl_3$) of the 2-phenylpyridine dearomatization reaction using **Pd@C$_{meso}$** (8 h, 55°C, 7% mol Pd) under a) 2 bar H_2, b) 2 bar D_2.

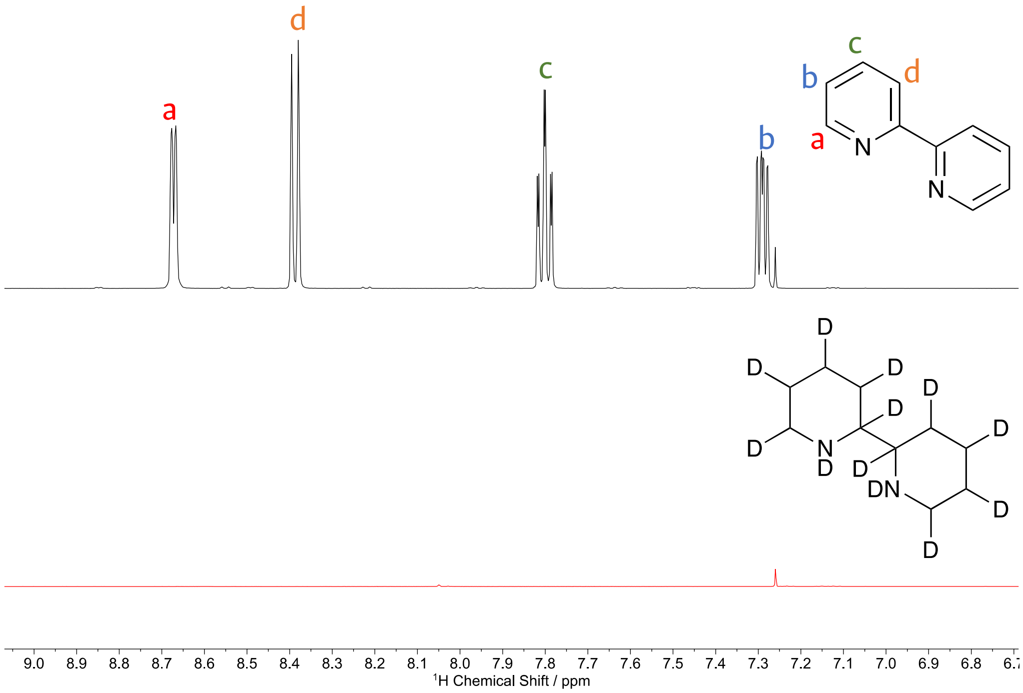

Figure S25. ^{1}H NMR spectrum (500 MHz, $CDCl_3$) for 2,2'-bipyridine (black) and 2,2'-bipyridine dearomatization (red) using **Pd@C$_{meso}$** (12 h, 70 ºC, 4 bar D_2, 7 mol % Pd).

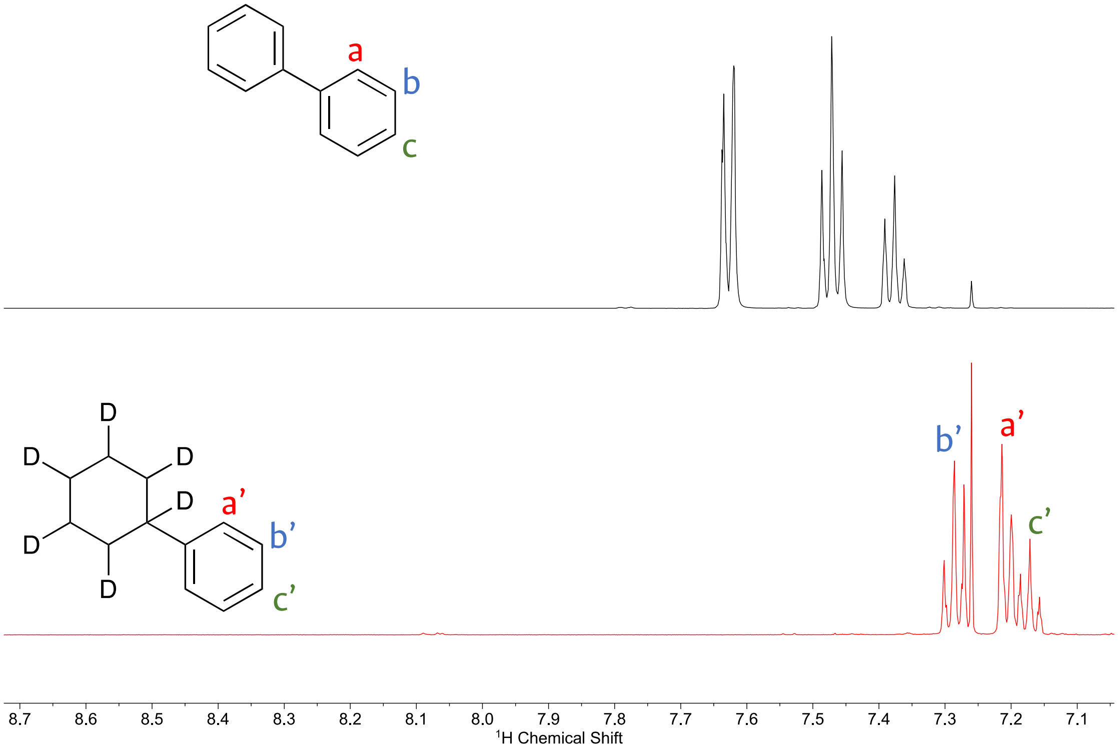

Figure S26. ^{1}H NMR spectrum (500 MHz, $CDCl_3$) for biphenyl (black) and biphenyl dearomatization (red) using **Pd@C$_{meso}$** (12 h, 70 ºC, 4 bar D_2, 7 mol % Pd).

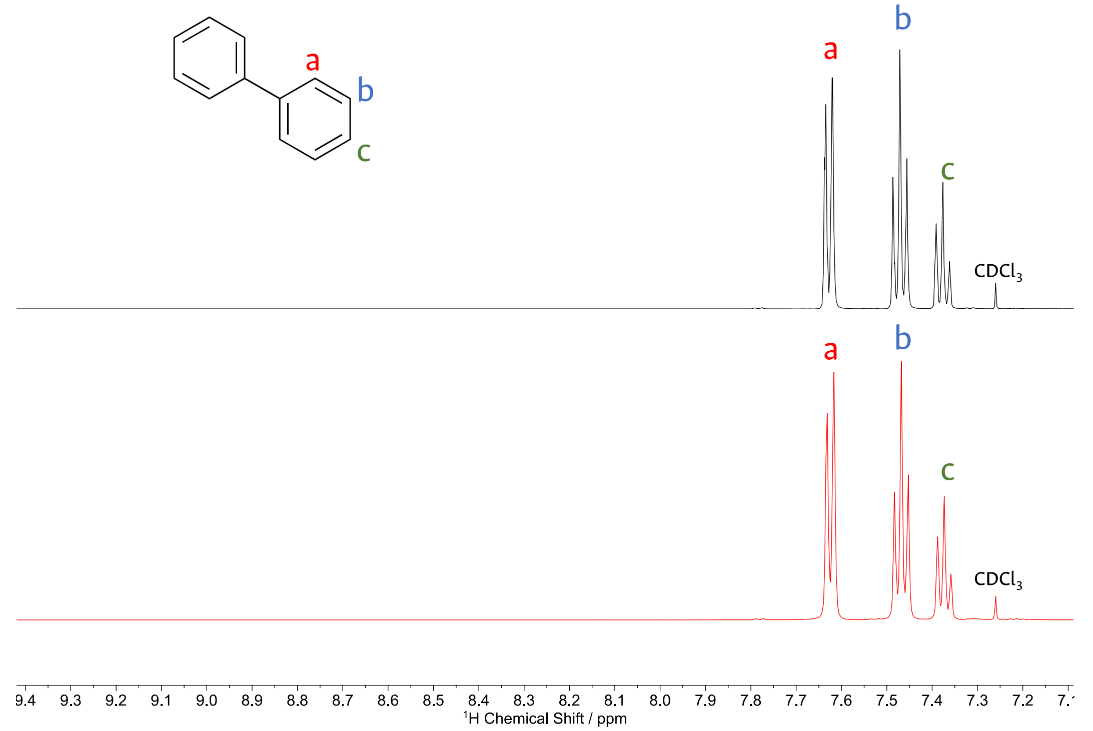

Figure S27. ^{1}H NMR spectrum (500 MHz, $CDCl_3$) for biphenyl (black) and biphenyl dearomatization (red) using **Pd@ICy$_{0.2}$** (17 h, 70 ºC, 4 bar D_2, 5 mol % Pd).

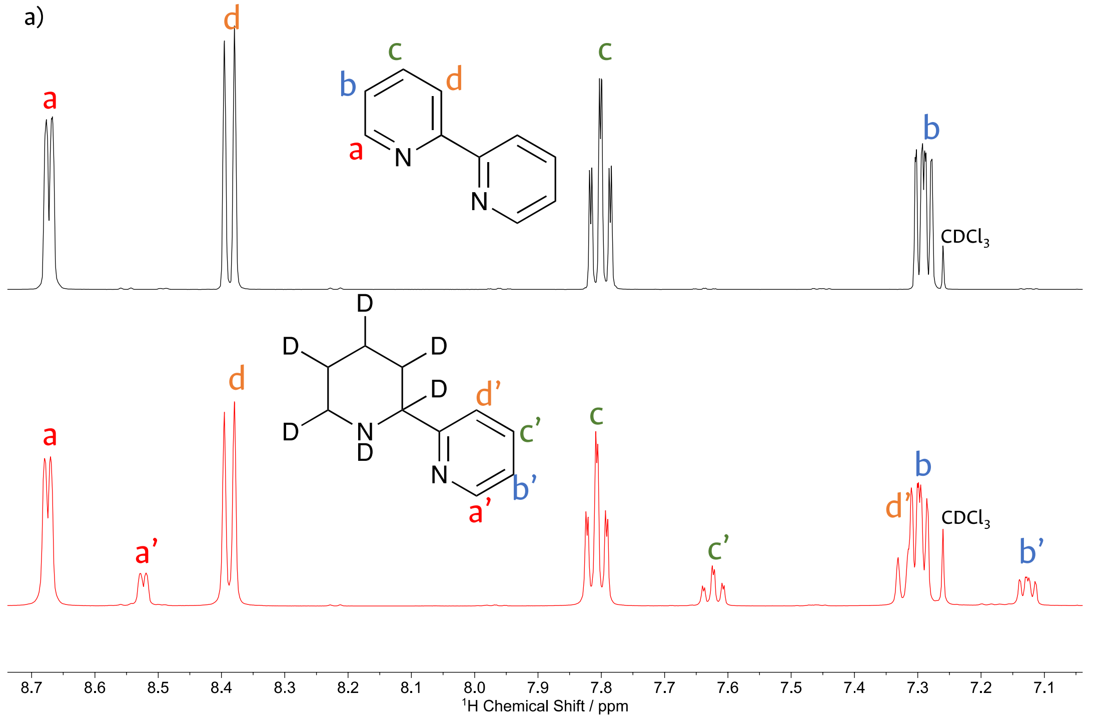

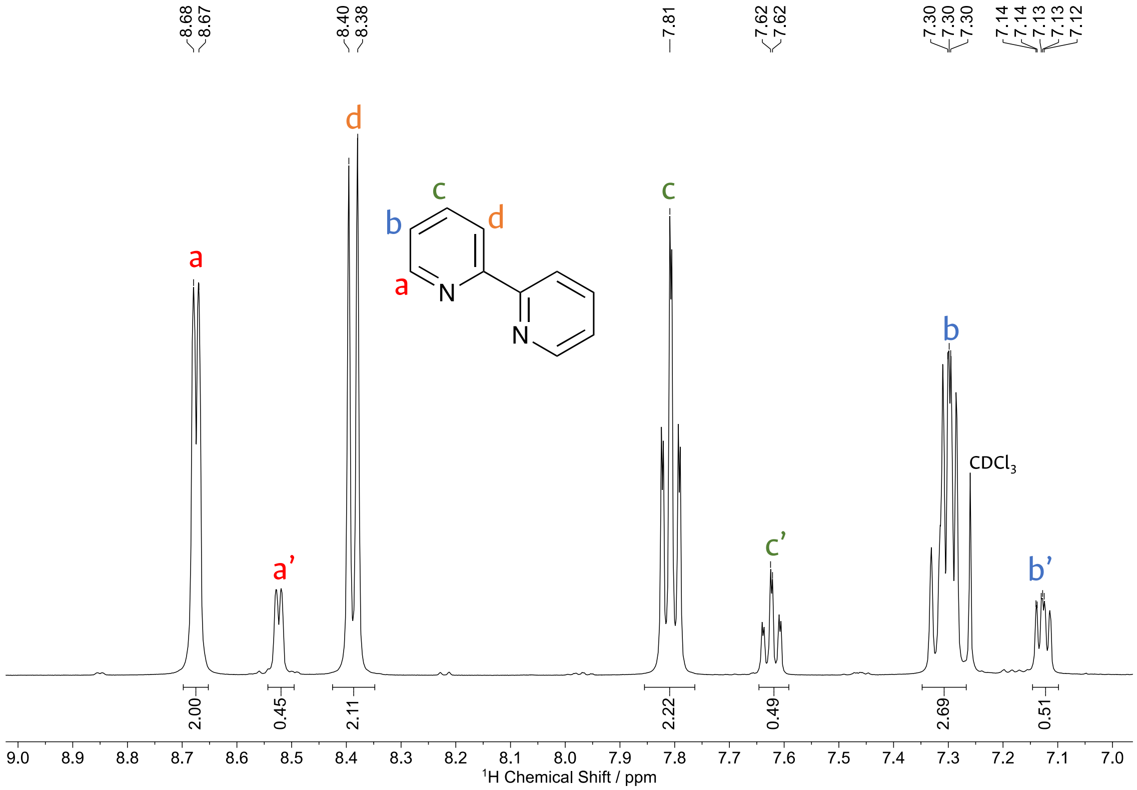

Figure S28. a) ^{1}H NMR spectrum (500 MHz, $CDCl_3$) for 2,2'-bipyridine (black) and 2,2'-bipyridine dearomatization (red) using **Pd@ICy$_{0.2}$** (17 h, 70 ºC, 4 bar D_2, 5 mol % Pd), b) integral values for 2,2'-bipyridine dearomatization with the same conditions previously mentioned.

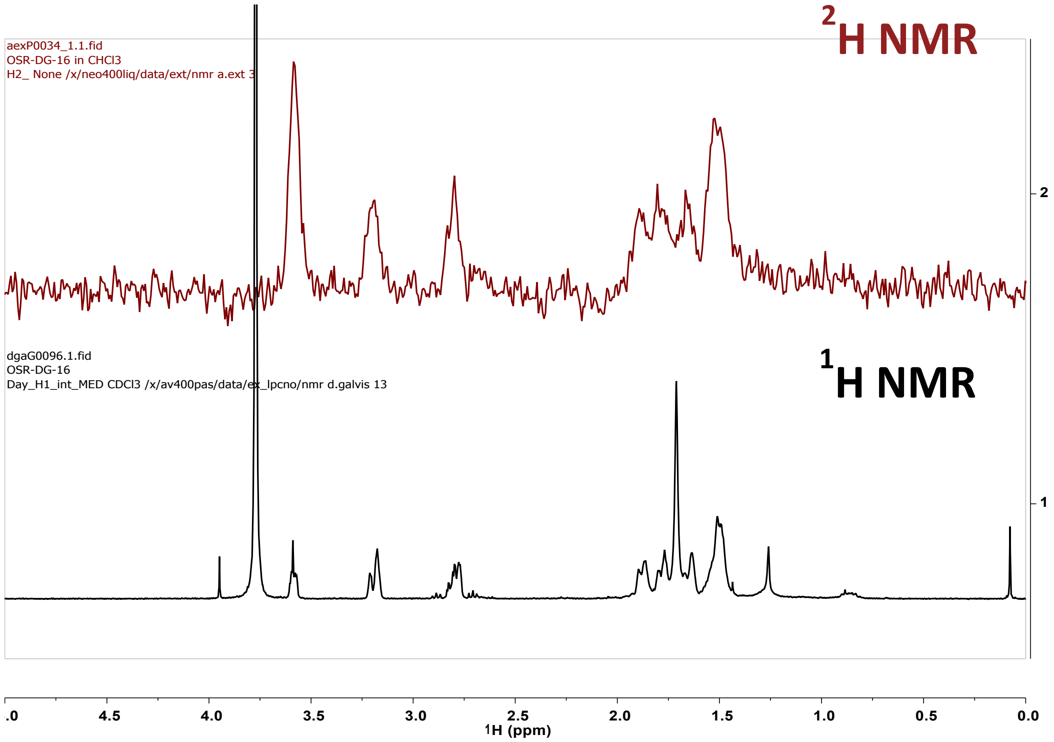

Figure S29. ^2H NMR (top, 77 MHz, $CHCl_3$) and ^1H NMR spectrum (down, 500 MHz, $CDCl_3$) for phenylpyridine reaction under a H_2/D_2 mixture using **Pd@C$_{meso}$** as a catalyst (8 h, 55 ºC, 2 bar total, 7 mol % Pd, using 1,3,5 trimethoxy benzene as internal standard)

11. Dearomatization reaction

Table S2. Dearomatization of 2-phenylpyridine using **Pd@C$_{meso}$** as a nanocatalyst.[a]

Entry	Time (h)	Temperature (ºC)	Pressure D_2 (bar)	Conversion[b] (%)
**1**	12	70	4	>99
**2**	12	70	4 (H_2)	>99
**3**	12	90	4	>99
**4**	12	90	4 (H_2)	>99
**5**	24	70	4	>99

**6**	24	70	4 (H_2)	>99
**7**	24	90	4	>99
**8**	24	90	4 (H_2)	>99

[a]7 mol% Pd (**Pd@C$_{\mathbf{meso}}$**), 0.1399 mmol 2-phenylpyridine, 5 mL THF.

[b]Determined by NMR.

12. DFT calculations

Choice of models

While nanoparticles exhibiting spherical, truncated octahedral, or cuboctahedral shapes - consistent with pure *fcc* bulk stacking - have been experimentally observed, alternative structures with different packing density, such as nanocubes, icosahedra, tetrahedra, and nanorods, have also been successfully synthesized and characterized.[15,16] On the computational side, the effect of hydrogen adsorption coverage on the shape of PdNPs has been investigated in a previous study by Cusinato and Hellman on several 55-atom models.[17] The relative stability of both ideal and defective nanoparticle morphologies was assessed using an *ab initio* thermodynamic phase diagram comparing various hydrogenated structures. The results showed that hydrogen adsorption impacts the relative stability of the nanoparticles, favoring icosahedron-based morphologies. This is in line with the well-known ability of metals such as Ag, Au, Cu, Pt, and Pd to form multiply twinned particles (MTPs).[18] It is commonly attributed to their low twinning energies, which allow them to relieve the strain associated with particles fully bounded by (111) facets.[19]

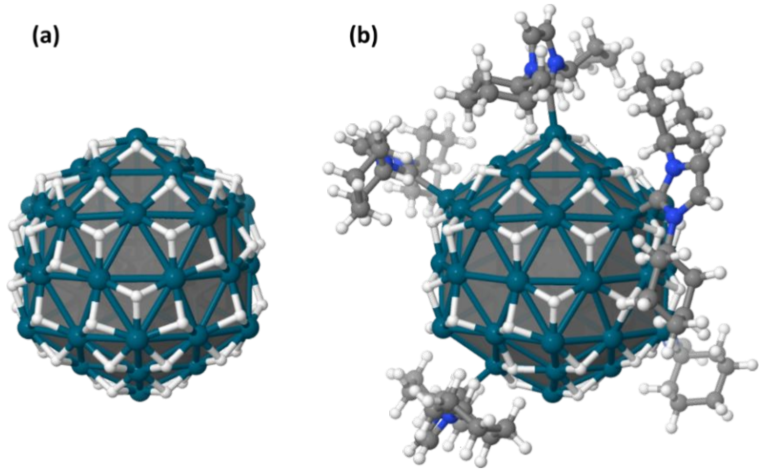

Figure S30. Representative models considered in this study for (a) **Pd@PVP**: $Pd_{55}(\mu_3\text{-}H)_{56}$; (b) **Pd@ICy$_{0.2}$**: $Pd_{55}(\mu_3\text{-}H)_{45}ICy_5$.

Physical origin of an inverse KIE

Let us first evaluate a possible inverse KIE arising from the standard physical origin, that lies in differences in zero-point vibrational energies (ZPVE). In the molecular case, namely in hydride vs. deuteride transfer reactions from transition metal hydrides to a substrate,[20] it has been shown that the overall change in zero-point energy between isotopologues (H *vs*. D) can lead to either a normal or inverse effect, depending on the shape of the potential energy surface and the character of the transition state and to possible "collective effects". It was summarized as the following rule of thumb: an inverse kinetic isotope effect ($k_H/k_D < 1$) is a result of the collective effects that involve that the relative ZPVE difference associated with C−H/C−D bond formation in the transition state being larger than that for M−H/M−D

bond cleavage. Formally, this corresponds to the inequality $\Delta_{CH/CD}{}^{‡} > \Delta_{MH/MD}$, where $\Delta_{XH/XD}$ = ZPVE(XH) - ZPVE(XD) and ‡ denotes the transition state of the transfer reaction. A conventional transition-state theory treatment of KIEs with the Born-Oppenheimer, rigid-rotor and harmonic oscillator approximations would necessitate investigating the full hydrogenation reaction pathway, an endeavor well beyond the primary scope of this work.

DFT calculations of metal nanoclusters.

Software: Vienna ab initio simulation package, VASP;[21,22] spin polarized DFT; exchange-correlation potential approximated by the generalized gradient approach proposed by Perdew, Burke, and Ernzerhof (PBE);[23] projector augmented waves (PAW) full-potential reconstruction;[24] PAW data sets for Ru treating the (n-1)p, (n-1)d and ns states (*i.e.* 18 valence electrons);[25] kinetic energy cutoff: 525 eV; Γ-centered calculations;[26] Gaussian smearing (σ) of 0.02 eV width, energies being therefore extrapolated for σ = 0.00 eV; geometry optimization threshold: residual forces on any direction less than 0.02 eV/Å; supercell size: 30×30.5×31 Å^3 (ensures a vacuum space of *ca*. 15 Å between periodic images of the nanoclusters).

Pd_{55} model. The model is an icosahedron.

Adsorption Energies

$$E_{ads}(H) = \frac{1}{n}\left[E(nH^*) - E(NP) - \frac{n}{2}E(H_2)\right]$$

$$E_{ads}(L) = \frac{1}{n}\left[E(nL^*) - E(NP) - \frac{n}{2}E(H_2)\right]$$

i.e. in the case of hydrides or deuterides it is a dissociative adsorption energy of H_2 or D_2.

Ab initio thermodynamics

Such a method has successfully been applied to explain or predict thermodynamic material properties,[27–29] and in particular surface properties at the solid–gas or, solid-liquid interface. In practice, it extends the T = 0 K and p = 0 Pa ab initio energies of surfaces covered by ligands coming from the surrounding medium to realistic environmental conditions in terms of temperature, pressure and composition of the gas or liquid phase. The adsorption process of a ligand L is simply: MNP + nL → nL* (MNP = metal nanoparticle and * stands for "chemisorbed") and the Gibbs free energy for this reaction is calculated as:

$$\Delta G_{ads}(T,nL) = \frac{\left[\Delta G^\circ - n\mu(L)\right]}{A}$$

Where A is the surface area of the MNP, μ is the chemical potential for L and ΔG° is calculated after DFT energies and vibrational contributions to energies. This methodology, also explained in detail in Ref[30] has been successfully applied by us to several cases.[31,32] The chemical potentials used in the present study for H_2 and D_2 are reported in Table S3. Calculated with the Gaussian16 software,[33] they show minor differences with those reported in the Janaf tables.[34] Generic H-Pd vibrational frequencies were calculated on Pd_{13} or Pd_{55} model covered with 1.2 H/Pd_{surf} (see Table S4). D-Pd harmonic frequencies were simply

deduced by dividing their Pd-H counterpart by 1.407, a value found after the Hooke's law and isotopic masses.

Table S3. Standard enthalpies and entropies used in this study

T/K	H_2		D_2	
	H(T,p°)/J.mol^{-1}	S(T,p°)/J.K^{-1}.mol^{-1}	H(T,p°)/J.mol^{-1}	S(T,p°)/J.mol^{-1}
**100.**	2999.0	100.73	2909.1	113.03
**200.**	5693.0	119.41	5820.7	133.20
**250.**	7089.0	125.64	7275.3	139.70
**298.15**	8467.0	130.68	8677.3	144.82
**300.**	8520.0	130.86	8729.8	145.00
**350.**	9969.0	135.33	10184.3	149.49
**400.**	11426.0	139.22	11641.5	153.37
**450.**	12887.0	142.66	13096.0	156.80
**500.**	14349.0	145.74	14555.8	159.87
**600.**	17278.0	151.08	17480.6	165.21
**700.**	20216.0	155.61	20431.6	169.76
**800.**	23169.0	159.55	23419.5	173.74
**900.**	26143.0	163.05	26449.3	177.31
**1000.**	29147.0	166.22	29526.4	180.56

Table S4. Calculated vibrational frequencies (cm^{-1}) for several coordination modes on the hydrogenated PdNP model.

coordination mode	Pd_{55} model
**μ-H**	1300 / 1150 / 280
**μ_3-H**	1050 / 1000 / 750
**μ_4-H**	650 / 450 / 400
**μ_6-H (Oh site)**	1050 / 425 / 375
**η^2-H_2**	3500 / 1000 / 675 / 300

The surface composition and energies considered for both models are reported in Table S5.

Table S5. Surface compositions and energies (in eV) considered for the calculation of the phase diagrams of $Pd_{55}X_n$ and $Pd_{55}X_nICY_5$ models (X= H or D). $Pd_{55}X_nICY_5$ models were selected after cheaper $Pd_{55}X_nICY_3$

preliminary calculations. Adsorption energies (ΔE_{ads}) and energy differences between isomers ($\Delta E_{isomers}$) are given in kcal/mol.

nH		E / eV	nH	ΔE_ads(H2)	ΔE_ads(n Icy)	ΔE_isomers	
	H2	-6.77					
	Icy	-240.63					
0	Pd55IC	-174.25	0				
0	Pd55IC-Icy3	-906.00	0		-75.92		
0	Pd55IC-Icy5	-1393.89	0		-76.17		
9	Pd55ICH09mu2	-210.44	9	-14.68			optimization converged to 9 mu3
17	Pd55ICH17mu2	-243.03	17	-15.25			optimization converged to 16 mu3, 1mu
33	IC-033H-041mu3	-307.78	33	-15.25			
33	IC-033H-041mu3-Icy3	-1038.38	33	-14.45	-67.03		
33	IC-033H-041mu3-Icy5	-1525.08	33	-13.62	-65.36	0.00	
39	IC-039Hmu3	-331.29	39	-14.80			
39	IC-039Hmu3-Icy3	-1061.58	39	-13.94	-64.69		
41	IC-041Hmu3	-339.22	41	-14.73			
41	IC-041Hmu3-Icy3	-1069.54	41	-13.92	-64.91		
41	IC-041Hmu3-Icy5	-1556.32	41	-13.30	-64.47	0.00	
41	IC-039Hmu3-2HOh	-339.22	41	-14.73			Oh were actually Td. Went out
41	IC-039Hmu3-2HOh-Icy3	-1069.50	41	-13.90	-64.64		Oh were actually Td. Went out
41	IC-039Hmu3-2HTRUEOh	-338.54	41	-14.35		15.65	HOh stayed in the Oh sites
41	IC-039Hmu3-2HTRUEOh-Icy3	-1068.76	41	-13.49	-64.15	17.92	HOh stayed in the Oh sites
45	IC-045Hmu3	-354.87	45	-14.50		0.00	
45	IC-045Hmu3-Icy3	-1084.81	45	-13.57	-61.97	0.00	
45	IC-045Hmu3-Icy5	-1571.57	45	-12.99	-62.59	0.00	
45	IC-039Hmu3-6HOh	-354.87	45	-14.50		0.02	none of the 6 HOh remained inside
45	IC-039Hmu3-6HOh-Icy3	-1084.59	45	-13.46	-60.26	5.16	The 6 HOh were actually Td. All went out
49	IC-049Hmu3	-369.50	49	-13.83			
49	IC-049Hmu3-Icy3	-1099.38	49	-12.95	-61.58		
52	IC-052Hmu3	-381.05	52	-13.65		0.00	
52	IC-052Hmu3-Icy3	-1110.52	52	-12.64	-58.37	0.00	
52	IC-052Hmu3-Icy5	-1596.85	52	-11.95	-58.45	0.00	
52	IC-052Hmu3_bis	-381.87	52	-14.01		-18.97	
52	IC-052Hmu3_bis-Icy3	-1111.15	52	-12.92	-63.26	-14.67	
52	IC-052Hmu3_bis-Icy5	-1597.32	52	-12.16	-56.84	-10.92	
52	IC-051Hmu3-1HOh	-380.76	52	-13.52		6.60	
52	IC-051Hmu3-1HOh-Icy3	-1110.27	52	-12.53	-58.64	5.80	
52	IC-051Hmu3-1HOh-Icy5	-1597.48	52	-12.23	-62.69	-14.61	a lot of mu3-H have changed of site on the surface
52	IC-51Hmu3-1Oh-AfterIcy5	-381.58	52	-13.89		-12.28	
52	IC-51Hmu3-1Oh-AfterIcy5-Icy3	-1111.19	52	-12.93	-59.42	-15.42	
52	IC-051Hmu3-1HOhsubH	-380.76	52	-13.52		6.59	
52	IC-051Hmu3-1HOhsubH-Icy3	-1110.27	52	-12.53	-58.66	5.72	
52	IC-048Hmu3-4Oh	-380.69	52	-13.49		8.35	
52	IC-048Hmu3-4Oh-Icy3	-1110.28	52	-12.53	-59.32	5.52	
56	IC-056Hmu3	-396.96	56	-13.65		0.00	
56	IC-056Hmu3-Icy3	-1126.23	56	-12.63	-56.82	0.00	
56	IC-056Hmu3-Icy5	-1612.08	56	-11.79	-55.30	0.00	
56	IC-055Hmu3-1HOh	-396.88	56	-13.62		1.88	
56	IC-055Hmu3-1HOh-Icy3	-1126.16	56	-12.60	-56.93	1.55	
56	IC-055Hmu3-1HOh-after56Hmu3-4HOh	-396.84	56	-13.60		2.84	
56	IC-055Hmu3-1HOh-after56Hmu3-4HOh-Icy3	-1126.12	56	-12.59	-56.95	2.45	
56	IC-055Hmu3-1HTdcore	-396.88	56	-13.62		1.87	Htd came back to the Oh site => idem IC-055Hmu3-1HOh
56	IC-055Hmu3-1HTdcore-Icy3	-1126.17	56	-12.60	-56.97	1.40	Htd came back to the Oh site => idem IC-055Hmu3-1HOh
56	IC-055Hmu3-1HTdcore-Icy5	-1612.18	56	-11.83	-56.12	-2.23	
56	IC-054Hmu3-002HOh	-396.71	56	-13.55		5.81	
56	IC-054Hmu3-002HOh-Icy3	-1126.14	56	-12.59	-58.04	2.14	
56	IC-054Hmu3-002HOh-Icy5	-1612.02	56	-11.76	-56.18	1.42	
56	IC-053Hmu3-002HOh-001HTd	-396.71	56	-13.55		5.81	2H Oh only (Htd moved to the surface)
56	IC-053Hmu3-002HOh-001HTd-Icy3	-1126.03	56	-12.55	-57.18	4.71	2H Oh, 1H Td (on the contrary to the no Icy case)
56	IC-053Hmu3-002HOh-001HTd-avec1Haudessus	-396.46	56	-13.44		11.68	
56	IC-053Hmu3-002HOh-001HTd-avec1Haudessus-Icy3	-1126.10	56	-12.57	-59.68	3.08	3H Oh
56	IC-045Hmu3-11HTd	-395.21	56	-12.93		40.53	
56	IC-045Hmu3-11HTd-Icy3	-1123.02	56	-11.31	-45.62	74.13	
56	IC-045H-5H2-1Hmu3	-394.32	56	-12.56		60.98	
56	IC-045H-5H2-1Hmu3-Icy3	-1124.22	56	-11.80	-61.68	46.40	
56	IC-054Hmu3-H2apx	-396.65	56	-13.52		7.30	remains H2
56	IC-054Hmu3-H2apx-Icy3	-1125.93	56	-12.50	-56.92	7.00	remains H2
56	IC-054Hmu3-H2edge	-396.43	56	-13.43		12.21	edge close to Icy
56	IC-054Hmu3-H2edge-Icy3	-1125.74	56	-12.43	-57.10	11.38	same as IC-052Hmu3-003HOh-001HTd-avec1Haudessus-Icy3, with 3 additional surface H
56	IC-52Hmu3-4Oh	-396.28	56	-13.37		15.85	
56	IC-52Hmu3-4Oh-Icy3	-1125.68	56	-12.40	-57.87	12.69	
56	IC-52Hmu3-4Oh-Icy5	-1611.95	56	-11.73	-57.86	3.07	
56	IC-056H-047mu2	-396.82	56	-13.59		3.31	Optimization converged to 52mu3H, 4muH
59	IC-056Hmu3-002HOh-001HTd-avec1Haudessus	-407.48	59	-13.10			
59	IC-056Hmu3-002HOh-001HTd-avec1Haudessus-Icy3	-1136.55	59	-12.05	-55.32		
60	IC-60Hmu3	-409.74	60	-12.45		0.00	
60	IC-60Hmu3-Icy3	-1139.24	60	-11.58	-58.61	0.00	
60	IC-60Hmu3-Icy5	-1625.69	60	-11.03	-59.16	0.00	
60	IC-59Hmu3-1Oh	-409.96	60	-12.53		-5.04	
60	IC-59Hmu3-1Oh-Icy3	-1139.84	60	-11.82	-61.58	-13.96	
60	IC-59Hmu3-1OhsubH	-409.96	60	-12.53		-5.03	
60	IC-59Hmu3-1OhsubH-Icy3	-1139.54	60	-11.70	-59.22	-6.87	
60	IC-58Hmu3-2HOh	-410.52	60	-12.75		-18.07	
60	IC-58Hmu3-2HOh-Icy3	-1139.95	60	-11.86	-58.08	-16.48	
60	IC-56Hmu3-4Oh	-411.06	60	-12.96		-30.45	
60	IC-56Hmu3-4Oh-Icy3	-1140.40	60	-12.03	-57.38	-26.75	
60	IC-56Hmu3-4Oh-Icy5	-1626.55	60	-11.36	-57.04	-19.86	
60	IC-54Hmu3-4HOh-1H2	-410.71	60	-12.82		-22.37	remains H2
60	IC-54Hmu3-4HOh-1H2-Icy3	-1140.03	60	-11.89	-57.22	-18.21	remains H2
60	IC-54Hmu3-6HOh	-410.90	60	-12.89		-26.71	
60	IC-54Hmu3-6HOh-Icy3	-1139.94	60	-11.85	-55.13	-16.28	
60	IC-53Hmu3-6HOh-1HTd	-410.90	60	-12.89		-26.71	Htd moved toward the surface
60	IC-53Hmu3-6HOh-1HTd-Icy3	-1139.85	60	-11.82	-54.42	-14.16	Htd remained in the Td site
67	IC-067H-071mu3-050Oh	-435.32	67	-11.80			
67	IC-067H-071mu3-050Oh-Icy3	-1164.57	67	-10.93	-56.68		
96	IC-096H-100mu3-H2067apx	-535.92	96	-8.82		0.00	
96	IC-096H-100mu3-H2067apx-Icy3	-1264.80	96	-8.13	-53.81	0.00	
96	IC-096H-2H2_convertisEn-HOh	-535.13	96	-8.63		18.25	
96	IC-096H-2H2_convertisEn-HOh-Icy3	-1263.96	96	-7.93	-53.47	19.26	
96	IC-096H-3H2-convertisEnHmu3HOh	-534.34	96	-8.44		36.55	
96	IC-096H-3H2-convertisEnHmu3HOh-Icy3	-1262.79	96	-7.65	-50.55	46.32	

Transition state (TS) search

Reaction barriers were estimated by the climbing image nudge elastic band (CINEB) method;[35] spring force between images: 5 eV; force tolerance of 0.02 eV Å. The harmonic

vibrational modes were systematically calculated to distinguish minima and saddle points by using the dynamical matrix code implemented in VASP as well as in-house tools.

Vibrational analysis

All intermediates and TS have been characterized by a normal mode analysis restricted to the involved reactant, TS and product (2-Phenylpyridine + H* or D*; hydrogenated 2-Phenylpyridine) as well as to the Pd active site. IRC-like explorations have been performed for all TSs.

Reaction energy profiles

Enthalpy values ($H°$), presented as an energy profile in the article, were calculated using the previously determined vibrational frequencies as input for ThermoWithVASP (an-in-house tool extensively validated on molecular systems, demonstrating identical enthalpy corrections to those obtained with Gaussian16). Vibrational analysis for deuterated systems was made by using the same POTCAR file as used for H, but with an atomic mass replacement.

Mechanistic study

The zero-point energy and thermal corrections to energies were also evaluated for H and D. ΔH° energies for D are reported in square brackets in **Figure 4** in the main text. **2PP** is quite strongly coordinated on the surface by *ca*. 20 kcal/mol. After its grafting on the Pd surface, **2PP*** reacts with a hydride (respectively deuteride) that changes from a μ_3 coordination to a terminal bonding in the transition state (**TS$_1$**), thus getting closer to the reacting carbon atom. The resulting singly hydrogenated compound, **C$_1$H***, is less stable than **2PP*** by ca. 27 kcal/mol, in relation with the decreased conjugation involved by the pyramidalization of the saturated C atom. As a result, the overall reaction is slightly endothermic. The geometries of **2PP***, **TS$_1$** and **C$_1$H***, shown in **Figure 4**, do not differ between the two surface coverage models. The comparison between H and D reveals no significant KIE, regardless of the surface composition. The main difference lies in the enthalpy of **C$_1$D***, which is more stable than its hydrogenated counterpart by 1 kcal/mol. However, this difference is insufficient to explain the observed KIE. The observed isotopic effect is more likely attributed to the higher surface coverage of deuterated PdNPs highlighted in the surface composition study. The red reaction profile plotted in **Figure 4** shows that, whereas the surface coverage does not significantly change the relative energies of **2PP*** and **C$_1$H***, the barrier height is divided by two (*ca*. 10 kcal/mol *vs*. 20 kcal/mol). This large difference could account for the apparent KIE on the dearomatization of 2-phenylpyridine observed with **Pd NPs.**

13. References

(1) Juhás, P.; Davis, T.; Farrow, C. L.; Billinge, S. J. L. *PDFgetX3* : A Rapid and Highly Automatable Program for Processing Powder Diffraction Data into Total Scattering Pair Distribution Functions. *J. Appl. Cryst.* **2013**, *46* (2), 560–566. https://doi.org/10.1107/S0021889813005190.

(2) Juhás, P.; Farrow, C. L.; Yang, X.; Knox, K. R.; Billinge, S. J. L. Complex Modeling: A Strategy and Software Program for Combining Multiple Information Sources to Solve Ill Posed Structure and Nanostructure Inverse Problems. *Acta Crystallogr A: Found. Adv.* **2015**, *71* (6), 562–568. https://doi.org/10.1107/S2053273315014473.

(3) Grimme, S.; Antony, J.; Ehrlich, S.; Krieg, H. A Consistent and Accurate Ab Initio Parametrization of Density Functional Dispersion Correction (DFT-D) for the 94 Elements H-Pu. *J. Chem. Phys.* **2010**, *132* (15), 154104. https://doi.org/10.1063/1.3382344.

(4) Bantreil, X.; Nolan, S. P. Synthesis of N-Heterocyclic Carbene Ligands and Derived Ruthenium Olefin Metathesis Catalysts. *Nat. Protoc.* **2011**, *6* (1), 69–77. https://doi.org/10.1038/nprot.2010.177.

(5) Amiens, C.; Chaudret, B.; Ciuculescu-Pradines, D.; Collière, V.; Fajerwerg, K.; Fau, P.; Kahn, M.; Maisonnat, A.; Soulantica, K.; Philippot, K. Organometallic Approach for the Synthesis of Nanostructures. *New J. Chem.* **2013**, *37* (11), 3374. https://doi.org/10.1039/c3nj00650f.

(6) Pieters, G.; Taglang, C.; Bonnefille, E.; Gutmann, T.; Puente, C.; Berthet, J.-C.; Dugave, C.; Chaudret, B.; Rousseau, B. Regioselective and Stereospecific Deuteration of Bioactive Aza Compounds by the Use of Ruthenium Nanoparticles. *Angew. Chem. Int. Ed. Engl.* **2014**, *53* (1), 230–234. https://doi.org/10.1002/anie.201307930.

(7) Martinez-Espinar, F.; Blondeau, P.; Nolis, P.; Chaudret, B.; Claver, C.; Castillón, S.; Godard, C. NHC-Stabilised Rh Nanoparticles: Surface Study and Application in the Catalytic Hydrogenation of Aromatic Substrates. *J. Cata.l* **2017**, *354*, 113–127. https://doi.org/10.1016/j.jcat.2017.08.010.

(8) Asensio, J. M.; Tricard, S.; Coppel, Y.; Andrés, R.; Chaudret, B.; de Jesús, E. Synthesis of Water-Soluble Palladium Nanoparticles Stabilized by Sulfonated N-Heterocyclic Carbenes. *Chem. Eur. J.* **2017**, *23* (54), 13435–13444. https://doi.org/10.1002/chem.201702204.

(9) Baquero, E. A.; Tricard, S.; Flores, J. C.; de Jesús, E.; Chaudret, B. Highly Stable Water-Soluble Platinum Nanoparticles Stabilized by Hydrophilic N-Heterocyclic Carbenes. *Angew. Chem. Int. Ed. Engl.* **2014**, *53* (48), 13220–13224. https://doi.org/10.1002/anie.201407758.

(10) Lara, P.; Rivada-Wheelaghan, O.; Conejero, S.; Poteau, R.; Philippot, K.; Chaudret, B. Ruthenium Nanoparticles Stabilized by N-Heterocyclic Carbenes: Ligand Location and Influence on Reactivity. *Angew. Chem. Int. Ed. Engl.* **2011**, *50* (50), 12080–12084. https://doi.org/10.1002/anie.201106348.

(11) Lara, P.; Suárez, A.; Collière, V.; Philippot, K.; Chaudret, B. Platinum N-Heterocyclic Carbene Nanoparticles as New and Effective Catalysts for the Selective

Hydrogenation of Nitroaromatics. *ChemCatChem* **2014**, *6* (1), 87–90. https://doi.org/10.1002/cctc.201300821.

(12) Pan, C.; Pelzer, K.; Philippot, K.; Chaudret, B.; Dassenoy, F.; Lecante, P.; Casanove, M.-J. Ligand-Stabilized Ruthenium Nanoparticles: Synthesis, Organization, and Dynamics. *J. Am. Chem. Soc.* **2001**, *123* (31), 7584–7593. https://doi.org/10.1021/ja003961m.

(13) Suárez-Riaño, O.; Mencia, G.; Tricard, S.; Esvan, J.; Fazzini, P.-F.; Chaudret, B.; Baquero, E. A. Water-Soluble NHC Pd/Ni Bimetallic Nanoparticles for H/D Exchange in Aromatic Amino-Acids. *Chem. Comm.* **2023**, *59* (8), 1062–1065. https://doi.org/10.1039/D2CC06019A.

(14) Cano, I.; Martínez-Prieto, L. M.; Fazzini, P. F.; Coppel, Y.; Chaudret, B.; van Leeuwen, P. W. N. M. Characterization of Secondary Phosphine Oxide Ligands on the Surface of Iridium Nanoparticles. *Phys. Chem. Chem. Phys.* **2017**, *19* (32), 21655–21662. https://doi.org/10.1039/C7CP03439C.

(15) Xiong, Y.; Xia, Y. Shape-Controlled Synthesis of Metal Nanostructures: The Case of Palladium. *Adv. Mat.* **2007**, *19* (20), 3385–3391. https://doi.org/10.1002/adma.200701301.

(16) Cheong, S.; Watt, J. D.; Tilley, R. D. Shape Control of Platinum and Palladium Nanoparticles for Catalysis. *Nanoscale* **2010**, *2* (10), 2045. https://doi.org/10.1039/c0nr00276c.

(17) Cusinato, L.; Hellman, A. Structure and Composition Modification of Ultrasmall Palladium Nanoparticles upon Hydrogenation from First Principles. *J. Phys. Chem. C* **2019**, *123* (30), 18609–18619. https://doi.org/10.1021/acs.jpcc.9b02724.

(18) Hofmeister, F. Fivefold Twinned Nanoparticles. In *Encyclopedia of Nanoscience and Nanotechnology*; Nalwa, H. S., Ed.; American Scientific Publishers, 2004; pp 431–452.

(19) Tao, A. R.; Habas, S.; Yang, P. Shape Control of Colloidal Metal Nanocrystals. *Small* **2008**, *4* (3), 310–325. https://doi.org/10.1002/smll.200701295.

(20) Cheng, T.-Y.; Bullock, R. M. Isotope Effects on Hydride Transfer Reactions from Transition Metal Hydrides to Trityl Cation. An Inverse Isotope Effect for a Hydride Transfer. *J. Am. Chem. Soc.* **1999**, *121* (13), 3150–3155. https://doi.org/10.1021/ja983448x.

(21) Kresse, G.; Furthmüller, J. Efficient Iterative Schemes for *Ab Initio* Total-Energy Calculations Using a Plane-Wave Basis Set. *Phys. Rev. B* **1996**, *54* (16), 11169–11186. https://doi.org/10.1103/PhysRevB.54.11169.

(22) Kresse, G.; Furthmüller, J. Efficiency of Ab-Initio Total Energy Calculations for Metals and Semiconductors Using a Plane-Wave Basis Set. *Comput. Mater. Sci.* **1996**, *6* (1), 15–50. https://doi.org/10.1016/0927-0256(96)00008-0.

(23) Perdew, J. P.; Burke, K.; Ernzerhof, M. Generalized Gradient Approximation Made Simple. *Phys. Rev. Lett.* **1996**, *77* (18), 3865–3868. https://doi.org/10.1103/PhysRevLett.77.3865.

(24) Blöchl, P. E. Projector Augmented-Wave Method. *Phys. Rev. B* **1994**, *50* (24), 17953–17979. https://doi.org/10.1103/PhysRevB.50.17953.

(25) Kresse, G.; Joubert, D. From Ultrasoft Pseudopotentials to the Projector Augmented-Wave Method. *Phys. Rev. B.* **1999**, *59* (3), 1758–1775. https://doi.org/10.1103/PhysRevB.59.1758.

(26) Monkhorst, H. J.; Pack, J. D. Special Points for Brillouin-Zone Integrations. *Phys. Rev. B.* **1976**, *13* (12), 5188–5192. https://doi.org/10.1103/PhysRevB.13.5188.

(27) Scheffler, M.; Stampfl, C. Theory of Adsorption on Metal Substrates. In *Handbook of Surface Science*; Elsevier, 2000; Vol. 2, pp 285–356. https://doi.org/10.1016/S1573-4331(00)80009-8.

(28) Arrouvel, C.; Digne, M.; Breysse, M.; Toulhoat, H.; Raybaud, P. Effects of Morphology on Surface Hydroxyl Concentration: A DFT Comparison of Anatase-TiO2 and γ-Alumina Catalytic Supports. *J. Catal.* **2004**, *222* (1), 152–166. https://doi.org/10.1016/j.jcat.2003.10.016.

(29) Reuter, K.; Scheffler, M. First-Principles Kinetic Monte Carlo Simulations for Heterogeneous Catalysis: Application to the CO Oxidation at Ru O_2 (110). *Phys. Rev. B – Condens. Matter. Mater. Phys.* **2006**, *73* (4). https://doi.org/10.1103/PhysRevB.73.045433.

(30) Cusinato, L.; Del Rosal, I.; Poteau, R. Shape, Electronic Structure and Steric Effects of Organometallic Nanocatalysts: Relevant Tools to Improve the Synergy between Theory and Experiment. *Dalton Trans*. **2017**, pp 378–395. https://doi.org/10.1039/c6dt04207d.

(31) del Rosal, I.; Truflandier, L.; Poteau, R.; Gerber, I. C. A Density Functional Theory Study of Spectroscopic and Thermodynamic Properties of Surfacic Hydrides on Ru (0001) Model Surface: The Influence of the Coordination Modes and the Coverage. *J. Phys. Chem. C* **2011**, *115* (5), 2169–2178. https://doi.org/10.1021/jp110090e.

(32) Rothermel, N.; Limbach, H.-H.; del Rosal, I.; Poteau, R.; Mencia, G.; Chaudret, B.; Buntkowsky, G.; Gutmann, T. Surface Reactions of Ammonia on Ruthenium Nanoparticles Revealed by ^{15}N and ^{13}C Solid-State NMR. *Catal. Sci. Technol.* **2021**, *11* (13), 4509–4520. https://doi.org/10.1039/D0CY02476G.

(33) Frissch, M. J.; Trucks, G. W.; Schlegel, H. B.; Scuseria, G. E.; Robb, M. A.; Cheeseman, J. R.; Scalmani, G.; Barone, V.; Petersson, G. A.; Nakatsuji, H.; Li, X.; Caricato, M.; Marenich, A. V.; Bloino, J.; Janesko, B. G.; Gomperts, R.; Mennucci, B.; Hratchian, H. P.; Ortiz, J. V.; Izmaylov, A. F.; Sonnenberg, J. L.; Williams-Young, D.; Ding, F.; Lipparini, F.; Egidi, F.; Goings, J.; Peng, B.; Petrone, A.; Henderson, T.; Ranasinghe, D.; Zakrzewski, V. G.; Gao, J.; Rega, N.; Zheng, G.; Liang, W.; Hada, M.; Ehara, M.; Toyota K.; Fukuda, R.; Hasewaga, J.; Ishida, M.; Nakajima, T.; Honda, Y.; Kitao, O.; Nakai, H.; Vreven, T.; Throssell, K.; Montgomery, J. A.; Peralta, J. E.; Ogliaro, F.; Bearpark, M. J.; Heyd, J. J.; Brothers, E. N.; Kudin, K. N.; Staroverov, V. N.; Keith, T. A.; Kobayashi, R.; Normand, J.; Raghavachari, K.; Rendell, A. P.; Burant, J. C.; Iyengar, S. S.; Tomasi, J.; Cossi, M.; Milliam, J. M.; Klene, M.; Adamo, C.; Cammi, R.; Ochterski, J. W.; Martin, R. L.; Morokuma, K.; Farkas, O.; Foresman, J. B.; Fox, D. J. Gaussian. Guassian, Inc.: Wallingford CT 2016.

(34) Chase Jr., M. W.; Davies, C. A.; Downey Jr, J. R.; Frurip, D. J.; McDonald, R. A.; Syverud, A. N. *JANAF Thermochemical Tables*; U.S. National Bureau of Standards, 1985.

(35) Henkelman, G.; Uberuaga, B. P.; Jónsson, H. A Climbing Image Nudged Elastic Band Method for Finding Saddle Points and Minimum Energy Paths. *J. Chem. Phys.* **2000**, *113* (22), 9901–9904. https://doi.org/10.1063/1.1329672.
